# The cost of illness and economic burden of endometriosis and chronic pelvic pain in Australia: A national online survey

**DOI:** 10.1371/journal.pone.0223316

**Published:** 2019-10-10

**Authors:** Mike Armour, Kenny Lawson, Aidan Wood, Caroline A. Smith, Jason Abbott

**Affiliations:** 1 NICM Health Research Institute, Western Sydney University, Sydney, New South Wales, Australia; 2 Translational Health Research Institute (THRI), Western Sydney University, Sydney New South Wales, Australia; 3 School of Women's and Children's Health, University of New South Wales, Sydney, New South Wales, Australia; Northwestern University, UNITED STATES

## Abstract

**Introduction:**

Endometriosis has a significant cost of illness burden in Europe, UK and the USA, with the majority of costs coming from reductions in productivity. However, information is scarce on if there is a differing impact between endometriosis and other causes of chronic pelvic pain, and if there are modifiable factors, such as pain severity, that may be significant contributors to the overall burden.

**Methods:**

An online survey was hosted by SurveyMonkey and the link was active between February to April 2017. Women aged 18–45, currently living in Australia, who had either a confirmed diagnosis of endometriosis via laparoscopy or chronic pelvic pain without a diagnosis of endometriosis were included. The retrospective component of the WERF EndoCost tool was used to determine direct healthcare costs, direct non-healthcare costs (carers) and indirect costs due to productivity loss. Estimates were extrapolated to the Australian population using published prevalence estimates.

**Results:**

407 valid responses were received. The cost of illness burden was significant in women with chronic pelvic pain (Int $16,970 to $ 20,898 per woman per year) irrespective of whether they had a diagnosis of endometriosis. The majority of costs (75–84%) were due to productivity loss. Both absolute and relative productivity costs in Australia were higher than previous estimates based on data from Europe, UK and USA. Pain scores showed the strongest relationship to productivity costs, a 12.5-fold increase in costs between minimal to severe pain. The total economic burden per year in Australia in the reproductive aged population (at 10% prevalence) was 6.50 billion Int $.

**Conclusion:**

Similar to studies in European, British and American populations, productivity costs are the greatest contributor to overall costs. Given pain is the most significant contributor, priority should be given to improving pain control in women with pelvic pain

## Introduction

Chronic pelvic pain (CPP) is pain in the pelvis of greater than six months duration, and is severe enough to cause functional disability or require medical intervention [1]. Common causes of chronic pelvic pain include endometriosis, adenomyosis, chronic infection, and functional disorders such as irritable bowel syndrome or interstitial cystitis. Endometriosis is the most common cause of CPP [2] with 24% to 40% of women with CPP having a diagnosis of endometriosis [3, 4] and whilst prevalence for CPP and endometriosis are variably quoted, all types of CPP range from 5.7% to 26.6% [5] of women. Real world estimates of endometriosis prevalence are between 5% [6] and 10% [7] of the reproductive aged female population.

A large multi-centre study across Europe, UK and the USA found that the total cost per woman with endometriosis per year was €9579 with the bulk of costs (€6298) being due to absence from work [8], with the economic burden of endometriosis being similar to or higher than other chronic disease burdens such as heart disease and diabetes [8]. Despite the majority of women with CPP not having a diagnosis of endometriosis [3, 4], there are few data on the cost of illness of non-endometriosis chronic pelvic pain. The economic impact may vary significantly between those with endometriosis and those with non-endometriosis related CPP due to the significant surgical interventions that often occur in endometriosis [9] however there is currently no economic analysis to support this hypothesis.

The aim of this survey and cost of illness (COI) analysis was to determine the economic impact of both women having chronic pelvic pain either with and without a current diagnosis of endometriosis on healthcare costs, employment related costs and other costs related to childcare and household maintenance for women in the Australian healthcare context. Whilst it is recognized that health and economic systems differ significantly between countries, even within close geographical areas [10], assessing the impact to the individual and at a societal level may help to guide policy and prioritisation for healthcare.

## Methods

### Survey

The WERF EndoCost tool was developed by the World Endometriosis Research Foundation (WERF) EndoCost Consortium, and the original protocol consists of validated prospective hospital questionnaires and both retrospective and prospective patient questionnaires [11]. Our study used the retrospective patient questionnaire component of the WERF EndoCost tool that was modified to an Australian demographic and healthcare context and hosted on SurveyMonkey (www.surveymonkey.com). The tool consisted of ninety-nine questions including direct healthcare costs (e.g. costs of medications and doctors visits), direct non-healthcare costs (e.g. transportation costs), and indirect costs of productivity loss. Total time to complete the survey was between 30–45 minutes. Modifications were made to income and ethnicity to adapt to Australian norms as per the Australian Bureau of Statistics [12]. Brand names for pharmaceuticals were modified to reflect their Australian brand names. The survey tool is available as S1 File. Analysis on other components of the WERF EndoCost tool (such as time to diagnosis, pelvic pain scores etc.) will be published separately.

The survey link was distributed via the social media platforms (Facebook, Twitter and Instagram) of Endometriosis Australia, EndoActive and Pelvic Pain Foundation of Australia. The total combined reach of these organisations on social media was just over 35k followers. Each organisation made two social media posts regarding the survey, the second post 3–5 weeks after the first. The survey link was active from February 2017 to April 2017, for a total of eight weeks. Data collection was closed once there had been no new responses for five days. Ethical approved was provided by the Western Sydney University Human Research Ethics Committee, approval number H12019.

Women were eligible to participate in the survey if they were aged 18–45, currently living in Australia and either had a surgically confirmed diagnosis of endometriosis, or if they had chronic pelvic pain from any cause. Chronic pelvic pain was defined as pain in the pelvis for at least six months that caused the woman to seek medical attention and if they had either; a laparoscopy that did not show evidence of endometriosis or had not undergone a laparoscopy at the time of survey. All data collected was from participants themselves. Following standard practice in cost-of-illness studies, this study measured costs rather than test a specific hypothesis and so no sample size calculation was necessary [11].

### Health care context

In Australia, there is a mixed public and private health care system. All residents have automatic access to the public system comprising: (i) primary (general practitioners (GP), allied health and selected pharmaceuticals), and (ii) secondary care (hospital, in-patient and out-patient). The government provides subsided care, and includes a co-payment mechanism resulting in patient out-of-pocket costs. The purchase of private insurance does not preclude using public hospitals. The assumption in this study was that patients visited public providers.

### Perspective

A ‘societal perspective’ was adopted, incorporating (i) health sector impacts, often termed ‘direct costs’, (ii) productivity impacts, often termed ‘indirect costs,’ using the human capital approach, incorporating multiplier impact [13] and (iii) household costs (out-of-pocket costs, and in-kind carer time costs). This was a prevalence study and estimated costs regardless of time of diagnosis, if known.

### Outcomes

The immediate aim was to estimate average per person costs, and separately, for (i) women with a diagnosis of endometriosis, and (ii) women reported suffering from general chronic pelvic pain, without an associated diagnosis. The average cost per person was estimated and for the age categories of 18–24, 25–30, 31–38 and 39+ years. Analysis was conducted by endometriosis and chronic pelvic pain separately. Mean and 95% confidence intervals were reported derived using bootstrapping with 5,000 replications [14]. An a priori decision was made to explore whether average costs differ by pain severity. The survey asked women to rate pain from 1–10, and then was collapsed into four categories: ‘Minimal’ (1–2), ‘Mild’ (3–5), ‘Moderate’ (6–8), ‘Severe’ (9–10). Women were then stratified, and the costing analysis as described above was repeated.

### Outcomes: Extrapolation to Australian population

Results from the survey were extrapolated by multiplying: (i) estimates of the prevalence of endometriosis using a rate of 10% [7], the most commonly accepted estimate (ii) the number of women in each age category (iii) average costs by age-category.

### Sensitivity analysis

An ‘analysis of extremes’ was conducted, where the key structural assumptions of the main analysis were altered to generate lower bound estimates. First, the Human Capital Approach was substituted with the Frictional Cost Approach where productivity impacts were capped at 3 months with the assumption of replacement in the workforce. Second, to account for potential uncertainty regarding unit costs estimates all estimates of costs and productivity estimates were lowered by 10%. Third, the population prevalence of endometriosis and CPP was then lowered to 5%.

Costs were estimated for one year and in AUD $ 2017 prices. Following standard practice, to enable comparison of the economic burden between countries, costs were converted to International dollars (Int $) using purchasing power parity (PPP) conversion factors so that Int $1 is equivalent to US $1 in the United States [15]. S2 File further details the methods used.

## Results

407 valid responses were received. 340 of these women had endometriosis (84%) and 67 (16%) had chronic pelvic pain without a current diagnosis of endometriosis (hereafter referred to just as CPP). Table 1 outlines the characteristics of the sample that were used in the costing analysis.

**Table 1:** Demographics of participants.

		ENDOMETRIOSIS		CHRONIC PELVIC PAIN		P VALUE
		Number	Percentage	Number	Percentage	
**AGE**	18–24	78	22.9%	16	23.9%	
** **	25–30	92	27.1%	15	22.4%	
** **	31–38	109	32.1%	27	40.3%	0.51
** **	39+	61	17.9%	9	13.4%	
** **	Total	340	100.0%	67	100.0%	
**PAIN SEVERITY**	Minimal	7	2.1%	3	4.5%	
** **	Mild	44	12.9%	11	16.4%	
** **	Moderate	87	25.6%	12	17.9%	0.22
** **	Severe	144	42.4%	31	46.3%	
** **	Unclassified	58	17.1%	10	14.9%	
**WEEKLY INCOME ($AUD)**						
** **	< $500	98	28.8%	16	23.9%	
** **	$501 to $1500	172	50.6%	37	55.2%	0.93
** **	$1501 to $3000	57	16.8%	11	16.4%	
** **	$3001 to $4500	6	1.8%	0	0.0%	
** **	> $4500	2	0.6%	1	1.5%	
** **	Did not state	5	1.5%	2	3.0%	

**Fig 1:**
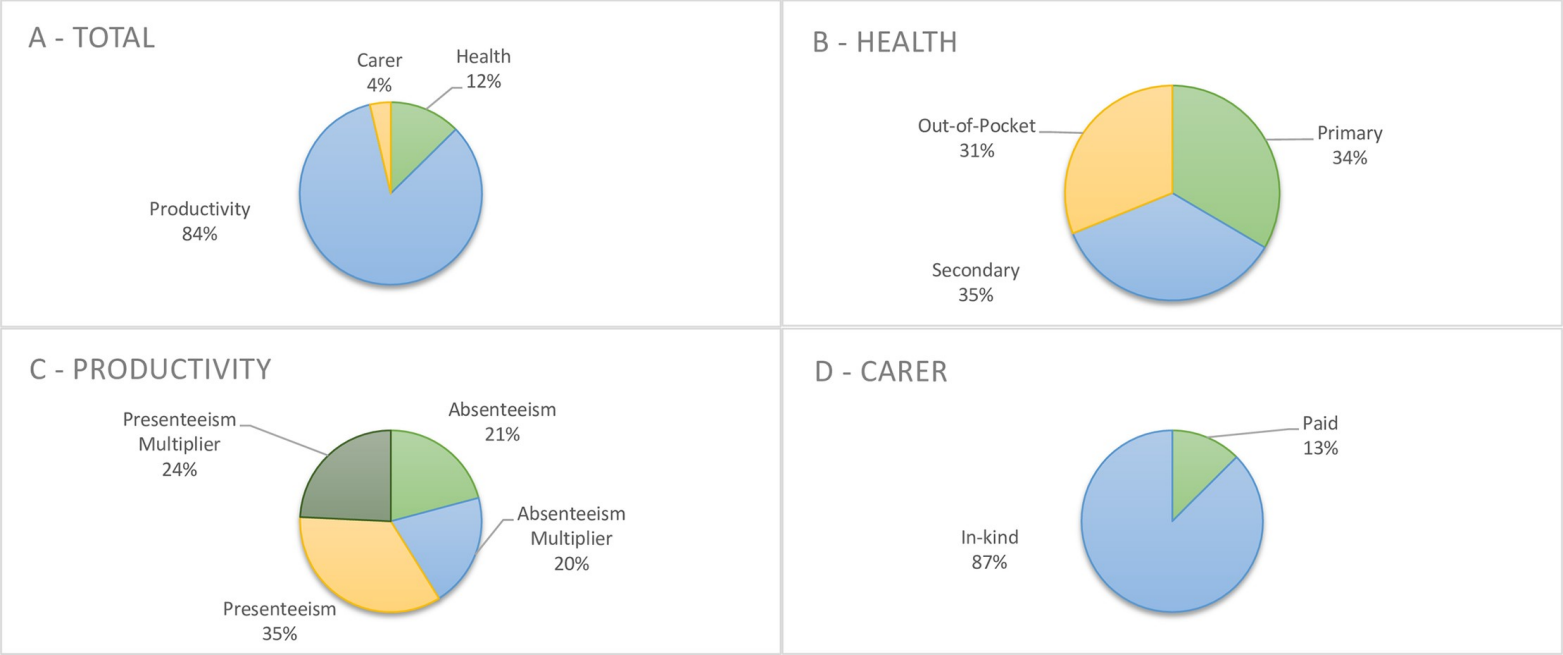
Cost breakdown for women with a diagnosis of endometriosis.

**Fig 2:**
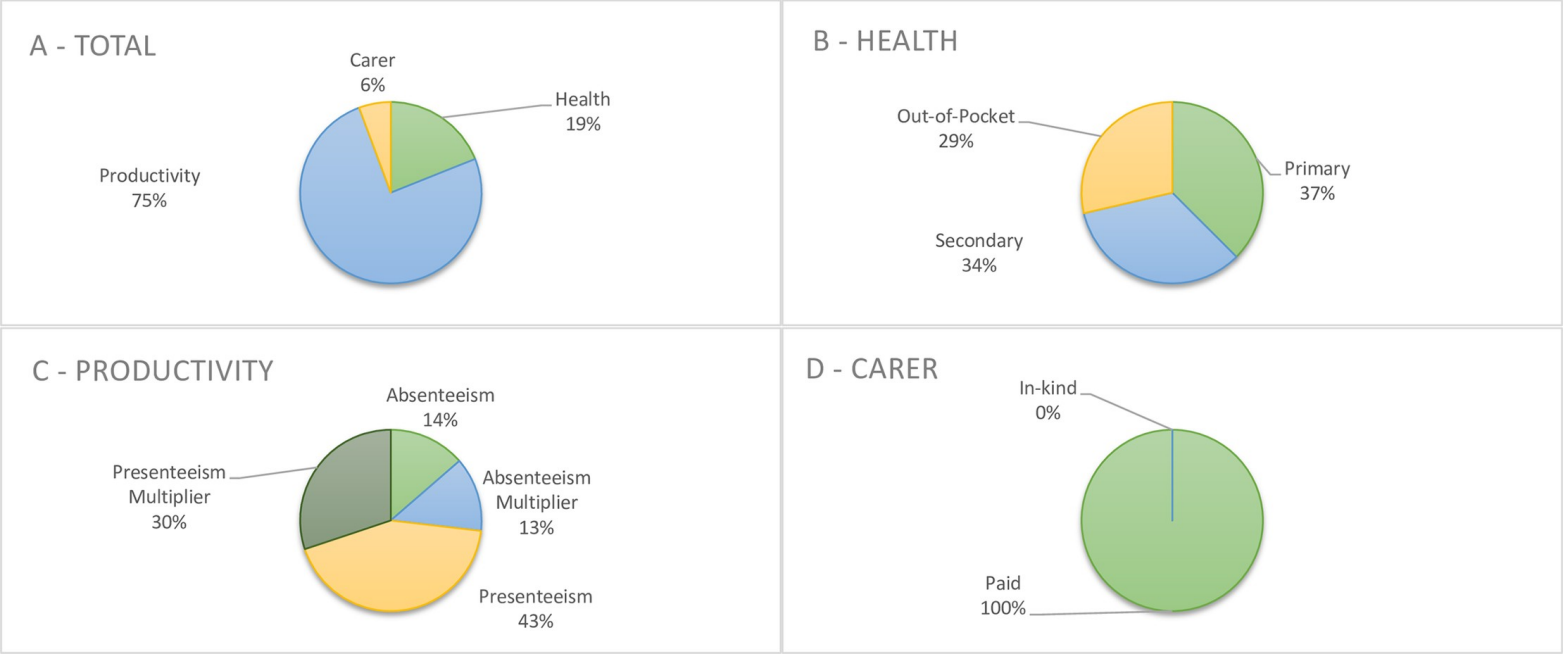
Cost breakdown for women with CPP.

The cost of illness was broken down into three categories; health related costs, productivity costs and cost for carers. The breakdown of costs in each category is outlined in Fig 1 for endometriosis and Fig 2 for CPP. Complete data for the cost breakdowns for both endometriosis and CPP is available in S1 Table. Total health related costs were relatively stable across all age groups and accounted for 12.5% and 19% of overall costs in endometriosis and CPP respectively. No patient reached the annual out of pocket fee limit of $1,521.8. The majority of the costs in both women with endometriosis and women with CPP were related to productivity costs, comprising 83.6% of total costs in women with endometriosis and 75% of total costs in women with CPP. Carer related costs were small, 3.8% of the total in women with endometriosis, and 5.7% in women with CPP.

For endometriosis, average per person, per year costs was estimated to be Int $ 2640 (95% CI 2158 to 3272) for total health costs, $17484 (95% CI 16407 to 18679) for productivity costs and $774 (95% CI 433 to 1262) for carer costs, for a total of $20,898 (95% CI 18,999 to 23,213) for all ages combined.

For women with CPP, average per person, per year costs was estimated to be Int $ 3215 (95% CI 2528 to 4234) for total health costs, $12,789 (95% CI 10,534 to 16,068) for productivity costs and $966 (95% CI 393 to 1499) for carer costs, for a total of $16,970 (95% CI 13,540 to 22,193) for all ages combined.

The major point of difference between the two cohorts were the social support structures; with women with endometriosis having mostly in-kind support (87%) with regards to carers, while women with CPP reported they did not receive any in-kind support, and all carer related costs were paid.

**Fig 3:**
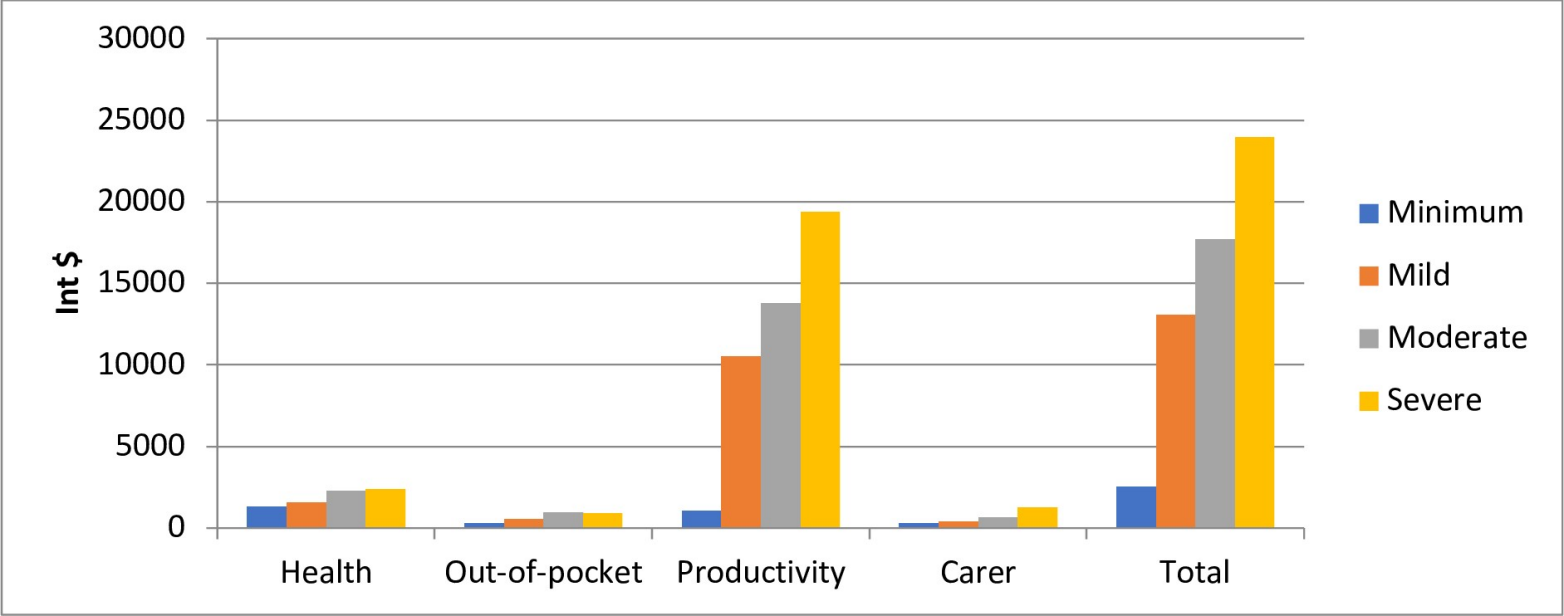
Costs broken down by pain severity (Int $).

Fig 3 and S2 Table outline costs by pain score in the women with endometriosis. The analysis was conducted for women suffering from endometriosis only, and for all age categories together. There were insufficient numbers to divide into age groups, or to repeat the analysis for chronic pelvic pain. Pain scores showed a strong relationship to overall cost ranging from Int $ 3,805, (95% CI 1617 to 5410) in women with minimal pain to Int $ 23987, (95% CI 21492 to 26774) in women with severe pain, over a 6-fold increase between minimal pain to severe pain. The magnitude of specific changes between minimal pain and severe pain were an approximately 2-fold increase in health-related costs and out of pocket costs, a 12.5-fold increase in productivity costs, and a 3-fold increase carer costs.

The total female population in Australia aged between 18–45 was 4.80 million in 2017 [16], and the total economic burden per year assuming a 10% prevalence in the reproductive aged population was 6.50 billion Int $.

In the sensitivity analyses, where lower bound estimates were generated the economic impacts fell substantially, as expected.

At a per person level, when a Frictional Cost Approach (FCA) was used (rather than the Human Capital Approach) and unit costs were reduced by 10%, then average per person costs were Int $ 8,116 (95% CI 7,056 to 8,877) for endometriosis and Int $ 8,563 (95% CI 7230 to 9765) for CPP. Regarding the population impact, if a prevalence rate of 5% (rather than 10%) was also used, then total costs were 1.37 billion Int $.

## Discussion

Women in Australia with endometriosis or other causes of CPP, have a substantial financial burden caused by their condition. We found the per person cost of endometriosis was Int $20,898 (95% CI 18,999 to 23,213) in 2017 prices. This is higher than previous work that also used the same tool where the estimated per person cost was Int $13,536, when converted to 2017 Int $[8]. Our study found lost productivity accounted for 83.6% of total costs in women with endometriosis and 75% of total costs in women with CPP, compared to 66–75% [8, 17] for women with endometriosis in previous work. We found a higher absolute and relative impact primarily because we included the ‘multiplier impacts’ when estimating productivity. This is an important economic consideration, with recent studies estimating these multiplier effects are important to incorporate in cost of illness studies [13, 18]. The proportionate sample size in this study was similar to previous reports of productivity suggesting comparability of these data between different health jurisdictions [8, 19].

The higher cost per patient found in our study may also reflect our recruitment strategy with participants drawn from social media engagement through major Australian endometriosis or pelvic pain support and advocacy groups [20]. Similar European studies [8, 17, 19] recruited from endometriosis clinics with women in our sample reporting significantly greater pain and work related impacts than clinic-based samples.

Importantly, the use of the WERF EndoCost tool in all of these surveys allows comparability and increases the body of evidence in this field. Our data reported not only on women surgically confirmed with endometriosis, but also with other forms of chronic pain–some of whom may be diagnosed with endometriosis in the future. It is important to recognize that women will present with ‘pain’ and not with ‘endometriosis’ and whilst we move to clinical diagnosis of endometriosis being more widely accepted[21], women will incur substantial costs no matter what their final diagnosis. Whilst there were differences in the cost of illness burden between the two cohorts we studied, especially in regard to carer support, these were small in terms of the absolute cost per woman per year. This is similar to previous work where pain intensity, and resulting costs, were similar between women with CPP and those with a diagnosis of endometriosis [22].

Our sensitivity analyses demonstrate that the economic burden varies from $1.4 billion (low) to $6.5 billion (high), mainly due to uncertainty regarding the true prevalence rates of endometriosis (5% or 10%), and the form of economic valuation regarding productivity impacts (HCA or the FCA). The HCA was used in the main analysis to represent the full impact on women affected by the conditions and was used by Simeons in a similar study [8]. The FCA approach caps the productivity impacts at 3 months assuming that women affected are replaced in the workforce and that the economy is no longer impacted. We know from the survey that women who reported being employed and affected by absenteeism/presenteeism were not actually replaced. Nonetheless, the FCA was included in the sensitivity analysis given this is a common approach to take. It has been recommended that all COI should include the multiplier impacts to capture the full productivity burden [18].

We chose to stratify cost of illness burden by pain, rather than ASRM disease stage, since the relationship between disease stage and pain is very poor [23–26]. Our findings showed that while all costs increased with increasing pain severity, productivity costs, both in percentage and absolute dollar value, were the most significant contributor to this. Previous research reports that women with endometriosis have greater productivity loss as pain increases [27] with chronic pain one of the most significant contributors to absenteeism or presenteeism in the work place [28]. Reasons for this include the difficulty that many women with CPP have for prolonged sitting (longer than 20 minutes) [29] the necessity to attend work, despite pain, due to having used all available sick leave [30]. The impacts of regular absenteeism or perceived low productivity can be wide ranging, with women reporting that these impacts ranged from ‘mild’ issues such as losing a chance for promotion, to having employment terminated or resignation due to stress [30].

Women with endometriosis report issues achieving adequate pain management [31] with NSAIDs and hormonal treatments offering limited efficacy, having problematic side effects [32], with discontinuation rates of between 25–50% [33]. The impact of pain reduction in improving productivity is an important consideration. Studies showing as little as a 10% reduction on a pain scale is needed [34] while the more generally accepted figure for chronic pain is 30% [35]. It is likely based on this that any reduction in pain greater than 10% may play a role in reducing productivity related costs and interventions for pain reduction should aim for clinically meaningful reductions that impact both quality of life for the woman and improved productivity with its benefits for both personal well-being and general productivity [27, 36].

### Strengths and limitations

Limitations of this study include the limited sample size, due in part to the in-depth nature of the questionnaires completed and information obtained. Whilst this is the first Australian study for endometriosis and there exist similar estimates from other countries, there are no previous studies on CPP. We asked women to answer the endometriosis section only if they had a histologically confirmed surgical diagnosis of endometriosis and confirmatory evidence is not possible due to the anonymous nature of the survey. Given the significant time and effort burden of filling in the survey (30–45 minutes) it’s unlikely that women without endometriosis would have undertaken this but we cannot rule this out. That would place them into the CPP group. Conversely, many women categorized as CPP may have undiagnosed endometriosis. Previous work suggests that the diagnosis of endometriosis may range from 33% [37] to 75% [38]. Importantly our study demonstrates a similar economic burden no matter which is the final diagnosis of CPP, and the key point is that both endometriosis and other forms of CPP result in substantial personal and societal economic impact.

Over 43% of participants had pain in the most severe range and without population level data on pain distribution, we cannot determine how representative our sample is of the larger endometriosis/CPP population and these data may over-represent those not responding well to medical or surgical management. We did not collect data on geographical location of respondents, so it is possible that our sample did not represent potential differences in the cost of illness that may exist between women in urban, rural and remote areas. However, based on our previous work in this population using the same recruitment strategies [39] we expect that both urban and rural women are well represented in this sample. Women in our survey reported an income in the $501–1500 AUD bracket—in line with the $1448AUD average weekly income (adjusted for the 0.89 gender pay gap) earned by adult Australian women in 2017 [40]. Finally, presenteeism and pain scores were estimated using a 7-day recall in keeping with the EndoCost tool. We recognize that pelvic pain scores, and by association presenteeism, fluctuate substantially during the month, often dependent on the phase of the menstrual cycle. Given the sample size it is likely that overall pain scores were represented at various stages of women’s menstrual cycles, and that the estimate for the population overall is representative of this cohort of sufferers. It is likely that the estimated health sector costs are an underestimate. The ENDCOST tool did not distinguish whether women accessed public or private care. The analysis assumed that all women used the public health system. All women would have used Medicare funded primary care, however this is unlikely for secondary care. Approximately 60% of adults over 18 years have a form of private insurance for secondary care[41], however it is unknown what percentage of women with endometriosis have insurance, whether this would cover relevant treatments and procedures, and the unit costs of private care are also not routinely available. Overall, the analysis is conservative.

## Conclusion

This research clarifies that endometriosis and chronic pelvic pain have considerable impact for the women affected; the health sector; the wider economy and to carers. Given the huge financial burden of endometriosis and CPP, there is an urgent need for accurate epidemiological studies to assess the true prevalence rate of endometriosis, and for substantive longitudinal studies that determine these economic impacts with greater accuracy to guide policy at a national and international level. The inclusion of economic evaluations alongside future intervention studies to assess cost effectiveness, will allow a greater understanding of how the economic burden is reduced, including improvements in quality of life for women suffering with endometriosis and other forms of CPP.

## Supporting information

S1 File Modified WERF ENDOCOST survey tool. (PDF)

S2 File Extended methods section. (PDF)

S3 File Deidentified survey data set. (XLSX)

S4 File Data dictionary. (DOCX)

S1 Table Cost breakdown by age and diagnosis. (PDF)

S2 Table Cost breakdown by pain category (Int $). (PDF)
