# Supplementary material for: The cost of illness and economic burden of endometriosis and chronic pelvic pain in Australia: A national online survey
Source: PLoS One. 2019 Oct 10;14(10):e0223316. doi: 10.1371/journal.pone.0223316 (PMC6786587; doi:10.1371/journal.pone.0223316)
Supplement: S1 File — (PDF) [file pone.0223316.s001.pdf]

Demographic information

1. How did you find out about this survey ?

- ○ from Endometriosis Australia (from web page, Facebook or other social media)
- ○ from Women's Health NSW
- ○ from the Pelvic Pain foundation of Australia
- ○ from the Australian Women’s Health Network
- ○ from ENDOACTIVE
- ○ from Endocenter
- ○ from my doctor / health professional
- ○ from family or friends
- ○ Other (please specify)

2. What is your date of birth

	DD		MM		YYYY
Date		/		/	

3. Highest level of education:

- ○ Primary school
- ○ Lower secondary (Year 10)
- ○ Upper secondary (Year 12)
- ○ Post-secondary, not university (i.e. TAFE or vocational college)
- ○ University
- ○ Postgraduate

4. Current relationship status:

- ◯ Single
- ◯ Married / De Facto (living with partner)
- ◯ In a relationship but not living with partner.
- ◯ Divorced / separated
- ◯ Widowed

5. Occupation: (please tick all that apply)

- ☐ Employee
- ☐ Self-employed
- ☐ Home duties / caring for children or family
- ☐ In education (going to school, university, etc.)
- ☐ Doing voluntary work
- ☐ Unable to work because of chronic pelvic pain symptoms
- ☐ Unable to work for other reasons
- ☐ Other (please specify)

6. Which of the following best describes your ethnicity:

- ◯ Caucasian
- ◯ Asian
- ◯ Aboriginal/TSI
- ◯ Polynesian
- ◯ Maori
- ◯ Unknown
- ◯ Other (please specify)

7. Do you have children ?

- ◯ Yes
- ◯ No

8. Income level - What is your average weekly earnings before tax

- < $500 per week
- $501 to $1500 per week
- $1501 to $3000 per week
- $3001 to $4500 per week
- > $4500 per week

Chronic pelvic pain

These questions relate to the chronic pelvic pain you have been currently or recently experiencing.

* 9. What is the cause of your chronic pelvic pain?

- ◯ Endometriosis (diagnosed via laparoscopy)
- ◯ Any other cause (including suspected endometriosis, chronic pelvic pain syndrome, or no known cause).

10. What year did you first experience symptoms of chronic pelvic pain?

[ ]

11. Which symptoms did you first experience? (please tick all that apply)

- [ ] Severe dysmenorrhoea (painful periods)
- [ ] Deep dyspareunia (pain on sexual intercourse)
- [ ] Pelvic pain
- [ ] Ovulation pain
- [ ] Cyclical or peri-menstrual symptoms (e.g. bowel or bladder associated) with or without abnormal bleeding
- [ ] Infertility
- [ ] Chronic fatigue

12. Year you first sought medical help for any of the above symptoms in**Q11**

[ ]

Endometriosis

13. What year did you get diagnosed with endometriosis ?

[ ]

14. How many doctors did you see before you were diagnosed with endometriosis?

[ ]

15. How many complementary therapists (e.g. acupuncturists, homeopaths, naturopaths) did you see before you were diagnosed with endometriosis?

[ ]

16. What stage was your endometriosis after your most recent laparoscopy (Stage I-IV)

- ◯ Stage I (minimal)
- ◯ Stage II (mild)
- ◯ Stage III (moderate)
- ◯ Stage IV (severe)
- ◯ I can't remember

Chronic pelvic pain (not diagnosed with endometriosis)

17. How many doctors have you seen to get a diagnosis for your chronic pelvic pain ?

18. If you have received a diagnosis for the cause of your chronic pelvic pain for your doctor, please write it below

19. How many complementary therapists have you seen for your chronic pelvic pain (e.g. acupuncturist, homeopath) ?

Pelvic pain

By ‘pelvic pain’ we mean any type of pain in the lower part of your belly (the area from your belly button down) to the area of the upper thighs.

* 20. In the **last 3 months,** have you had pelvic pain with your periods?

◯ Yes

◯ No

Pelvic pain with periods (dysmenorrhea)

21. How often have you had pelvic pain with your periods in the **last 3 months**?

- ◯ Occasionally (with 1 of my last 3 periods)
- ◯ Often (with 2 in 3 of my last 3 periods)
- ◯ Always (with all of my last 3 periods)

22. In the **last 3 months**, have you taken pain-killers for the pain that are prescribed for you by a **doctor**?

- ◯ No
- ◯ Yes

23. **In the last 3 months**, have you taken pain-killers for the pain, bought **over the counter** without prescription?

- ◯ Yes
- ◯ No

24. **In the last 3 months**, has your period pain prevented you from going to work or carrying out your daily activities (even if taking pain-killers) ?

- ◯ Never
- ◯ Occasionally (in 1 of my last 3 periods)
- ◯ Often (in 2 of my last 3 periods)
- ◯ Always (in all of my last 3 periods)

25. **In the last 3 months**, have you had to lie down for any part of the day or longer because of your period pain?

- ◯ Never
- ◯ Occasionally (in 1 of my last 3 periods)
- ◯ Often (in 2 of my last 3 periods)
- ◯ Always (in 3 of my last 3 periods)

26. Please circle on the following scale, going from no pain (0) to worst possible pain (10), the number that indicates how severe your period pain has been **on average in the last 3 months**:

0 10

27. Please circle on the following scale, going from no pain (0) to worst possible pain (10), the number that indicates how severe your period pain has been **at its worst in the last 3 months**:

0 10

28. The following questions are about your bowel movements/stool when you had period pain**in the last 3 months**

	Never/Rarely	Sometimes	Often	Most of the time	Always
how often did your period pain get better or stop after you had a bowel movement?					
how often did you have more frequent bowel movements?					
how often did you have less frequent bowel movements?					
were your stools (bowel movements) looser?					
were your stools (bowel movements) harder ?					

The following questions are around how your period pain affected you at different ages. If you haven't reached an age range yet, for example if you are 26 please just leave the 31-35 years and the 36 years and over sections blank.

29. On average, how often did you have painful periods at each of these age groups ?

	Never	Occasionally (with less than a quarter of my periods)	Often (with a quarter to half my periods)	Usually (with more than half my periods)	Always (with every period)	Can't remember
up to 20 years						
21-25 years						
26-30 years						
31-35 years						
36 years and over						

30. Did you take pain-killers prescribed for you by a doctor for your period pain at each of these age groups ?

	Yes	No
up to 20 years	◯	◯
21-25 years	◯	◯
26-30 years	◯	◯
31-35 years	◯	◯
36 years and over	◯	◯

31. Did you take pain-killers that you bought over the counter (*without prescription*) at of these age groups ?

	Yes	No
up to 20 years	◯	◯
21-25 years	◯	◯
26-30 years	◯	◯
31-35 years	◯	◯
36 years and over	◯	◯

32. Did your period pain prevent you from **going to work or carrying out your daily activities** (even if taking pain-killers) ?

	Never	Occasionally	Often	Always	Can't remember
up to 20 years	◯	◯	◯	◯	◯
21-25 years	◯	◯	◯	◯	◯
26-30 years	◯	◯	◯	◯	◯
31-35 years	◯	◯	◯	◯	◯
36 years and over	◯	◯	◯	◯	◯

33. Did you have to **lie down** for any part of the day or longer because of your period pain?

	Never	Occasionally	Often	Always	Can't remember
up to 20 years	◯	◯	◯	◯	◯
21-25 years	◯	◯	◯	◯	◯
26-30 years	◯	◯	◯	◯	◯
31-35 years	◯	◯	◯	◯	◯
36 years and over	◯	◯	◯	◯	◯

Pain during or after sexual intercourse

The following questions relate to pain either during or after sexual intercourse. All information will be treated in complete confidence and is anonymous.

You have an option below to skip these questions if you do not wish to answer them.

34. In the **last three months** have you had pelvic pain **during or in the 24 hours after sexual intercourse?**

- ◯ Not applicable: I have not had sexual intercourse in the last 3 months
- ◯ No
- ◯ Yes
- ◯ I don't wish to answer these questions (skip to next section)

Pain during or after sexual intercourse

35. On average, **how often** do you have pelvic pain during or in the 24 hours after intercourse?

- ○ Never
- ○ Occasionally (less than a quarter of the times)
- ○ Often (a quarter to half of the times)
- ○ Usually (more than half of the times)
- ○ Always (every time)
- ○ Can't remember

36. Do you ever **interrupt** intercourse because of pelvic pain?

- ○ Yes
- ○ No

37. Do you ever **avoid** intercourse because of pelvic pain?

- ○ Yes
- ○ No

38. Is there a time of the month in which intercourse is **more painful** than at other times? (please select all that apply)

- ☐ No
- ☐ Yes: during a period
- ☐ Yes: just before or after a period
- ☐ Yes: at mid-cycle (around ovulation)
- ☐ Other (please specify)

____

39. Please circle on the following scale, going from no pain (0) to worst possible pain (10), the number that indicates how severe your pain **during sexual intercourse** has been on average in the **last 3 months**:

0 ———————————— 10 [ ]

40. Please circle on the following scale, going from no pain (0) to worst possible pain (10), the number that indicates how severe your pain **in the 24 hours after** sexual intercourse was on average in the **last 3 months:**

0 10

Other pelvic pain

* 41. In the last 3 months, have you had pelvic pain at times**OTHER** than when you have your period or during/after intercourse?

- ◯ Yes
- ◯ No

Non-menstrual pelvic pain

These questions relate to pelvic pain that you have which doesn't occur during your period OR during sexual intercourse

42. How long ago did this pain first start?

- ◯ 0 to 3 months ago
- ◯ 4-6 months ago
- ◯ 7-12 months ago
- ◯ Between 1 and 5 years ago
- ◯ If more than 5 years ago, please enter number of years

[ ]

43. Do you usually have this pain **at about the same time in your cycle?**

- ◯ No
- ◯ Yes, just before a period
- ◯ Yes, just after a period
- ◯ Yes, at mid-cycle (ovulation)

44. Approximately **how long in total** did you have this pain for in the **last 3 months?**

- ◯ Less than one day a month
- ◯ One day a month
- ◯ 2-3 days a month
- ◯ One day a week
- ◯ More than one day a week
- ◯ Every day

45. Do you take pain-killers for this pain, **prescribed for you by a doctor**?

- ◯ Yes
- ◯ No

46. Do you take pain-killers for this pain **that you can buy without a prescription?** (e.g. Aspirin, Nurofen, Paracetamol)

- Yes
- No

47. Please circle on the following scale, going from no pain (0) to worst possible pain (10), the number that indicates how severe your pain at times **OTHER than with periods or intercourse** has been ***on average*** in the **last 3 months**:

0 10

48. Please circle on the following scale, going from no pain (0) to worst possible pain (10), the number that indicates how severe your pain at times **OTHER than with periods or intercourse** has been at its ***worst*** in the **last 3 months**:

0 10

49. Please circle on the following scale, going from no pain (0) to worst possible pain (10), the number that indicates how severe your pain was **AT ITS WORST during your last internal gynaecological examination**:

0 10

Overall Health

The following questions ask for your views about your health and how you feel about life in general. If you are unsure about how to answer any question, try and think about your overall health and give the best answer you can. Do not spend too much time answering as your immediate response is likely to be the most accurate.

50. In general, would you say your health is:

- ◯ Excellent
- ◯ Very good
- ◯ Good
- ◯ Fair
- ◯ Poor

51. Compared to **one year ago**, how would you rate your health in general**now**?

- ◯ Much better than one year ago
- ◯ Somewhat better than one year ago
- ◯ About the same
- ◯ Somewhat worse now than one year ago
- ◯ Much worse now than one year ago

52. The following questions are about activities you might do during a typical day.
Does your health limit you in these activities?
If so, how much?

	Yes, Limited a lot	Yes, Limited a little	No, not limited at all
**Vigorous activities**, such as running, lifting heavy objects, participating in strenuous sports	◯	◯	◯
**Moderate activities**, such as moving a table,pushing a vacuum, bowling or playing golf	◯	◯	◯
**Lifting** or carrying groceries	◯	◯	◯
Climbing **several** flights of stairs	◯	◯	◯
Climbing **one** flight of stairs	◯	◯	◯
**Bending**, kneeling or stooping	◯	◯	◯
Walking **more than one kilometre**	◯	◯	◯
Walking **several hundred meters**	◯	◯	◯
Walking **100 meters**	◯	◯	◯
Bathing and dressing yourself	◯	◯	◯

53. **During the past 4 weeks**, how much of the time have you had any of the following problems with your work or other regular daily activities **as a result of your physical health**?

	All of the time	Most of the time	Some of the time	A little of the time	None of the time
Cut down on the **amount of time** you spent on work and other activities	◯	◯	◯	◯	◯
**Accomplished less** than you would like	◯	◯	◯	◯	◯
Were limited in the **kind** of work/activities	◯	◯	◯	◯	◯
Had difficulty performing the work or other activities (e.g. it took more effort)	◯	◯	◯	◯	◯

54. **During the past 4 weeks**, how much of the time have you had any of the following problems with your work or other regular daily activities **as a result of any emotional problems** (such as feeling depressed or anxious)?

	All of the time	Most of the time	Some of the time	A little of the time	None of the time
Cut down on the **amount of time** you spent on work and other activities	◯	◯	◯	◯	◯
**Accomplished less** than you would like	◯	◯	◯	◯	◯
Did work or other activities **less carefully** than usual	◯	◯	◯	◯	◯

55. **During the past 4 weeks**, to what extent have your physical health or emotional problems interfered with your normal social activities with family, neighbours or groups?

◯ Not at all

◯ Slightly

◯ Moderately

◯ Quite a bit

◯ Extremely

56. How much **bodily pain** have you had during the **past 4 weeks**?

◯ None

◯ Very mild

◯ Mild

◯ Moderate

◯ Severe

◯ Very severe

57. **During the past 4 weeks**, how much did **pain** interfere with your normal work (including both outside the home and housework)?

◯ Not at all

◯ Slightly

◯ Moderately

◯ Quite a bit

◯ Extremely

58. How much time during the last 4 weeks…..:

	All of the time	Most of the time	Some of the time	A little of the time	None of the time
Did you feel full of life?	○	○	○	○	○
Have you been very nervous?	○	○	○	○	○
Have you felt so down in the dumps that nothing would cheer you up?	○	○	○	○	○
Have you felt calm and peaceful?	○	○	○	○	○
Did you have a lot of energy?	○	○	○	○	○
Have you felt downhearted and low?	○	○	○	○	○
Did you feel worn out?	○	○	○	○	○
Have you been happy?	○	○	○	○	○
Did you feel tired?	○	○	○	○	○

59. **During the past 4 weeks**, how much of the time has your**physical health or emotional problems** interfered with your social activities (like visiting friends, relatives, etc)

- ○ All of the time
- ○ Most of the time
- ○ Some of the time
- ○ A little of the time
- ○ None of the time

60. How true or false are the following statements for you

	Definitely true	Mostly true	Not sure	Mostly false	Definitely false
I seem to get ill more easily than other people	○	○	○	○	○
I am as healthy as anybody I know	○	○	○	○	○
I expect my health to get worse	○	○	○	○	○
My health is excellent	○	○	○	○	○

Medical history

61. From the list below please mark whether you have had any of the following medical conditions

- ☐ Asthma
- ☐ Breast Cancer
- ☐ Chronic Fatigue Syndrome (M.E.)
- ☐ Deafness or difficulty hearing
- ☐ Depression requiring medication or medical consultation
- ☐ Diabetes requiring insulin or tablets
- ☐ Eczema
- ☐ Fibroid uterus
- ☐ Fibromyalgia
- ☐ Glandular Fever
- ☐ Hashimoto's disease
- ☐ Incomplete opening of the vagina (imperforate hymen)
- ☐ Lymphoma – Hodgkin's
- ☐ Lymphoma – Non-Hodgkin's
- ☐ Melanoma
- ☐ Multiple Sclerosis
- ☐ Ovarian Cancer
- ☐ Ovarian Cysts (benign)
- ☐ Polycystic Ovary Syndrome
- ☐ Pyloric Stenosis
- ☐ Rheumatoid Arthritis
- ☐ Scoliosis (curvature of the spine)
- ☐ Other spine problems
- ☐ Sjogren's syndrome
- ☐ Thyroid disease
- ☐ Mitral valve prolapse
- ☐ SLE (Lupus)
- ☐ Migraine
- ☐ Infertility
- ☐ Other (please specify)

62. Check whether or not any of the following conditions have occurred among blood relatives. If a condition has occurred, please indicate which relative(s) had/have the condition.

	Mother	Sister	Grandmother or Aunt on Mother's side	Grandmother or Aunt on Father's side
Endometriosis				
A double or divided uterus				
Menopause before aged 46 (not due to hysterectomy)				

Surgical procedures

Some women with pelvic pain, especially if caused by endometriosis, can experience fertility issues. Please provide details of the following hospital surgical procedures that you have received because of pelvic pain and/or infertility during your life up until the day you complete this survey

63. Type of procedure. Please enter the number of each type of procedure you have had. If you haven't had the procedure performed, please enter 0

Laparoscopy (key hole surgery)

Laparotomy (open surgery)

64. If you had either procedure in the previous question, please indicate the number of each type of procedure. If you did not have that procedure please enter 0

Diagnosis and/or removal of endometriosis

Removal of one ovary or both ovaries (via keyhole surgery)

Removal of one ovary or both ovaries (via open surgery)

Removal of uterus/womb (via key hole surgery)

Removal of uterus/womb (via open surgery)

Removal of uterus/womb and ovaries in the same procedure (via key hole surgery)

Removal of uterus/womb and ovaries in the same procedure (via open surgery)

Fertility treatment

Please enter the number of cycles of the followingfertility treatments that you received during your life up until the day you completed this survey. Please enter ‘0’ if you did not receive any cycles.

65. Please enter how many cycles of each of the following treatments you have had. If none please enter '0'

Treatment with hormonal stimulation using pills (Clomid, Nolvadex, Arimidex, Aromasin, Femara, etc.) combined with spontaneous sexual intercourse (without intrauterine insemination)

Treatment with hormonal stimulation using injections of gonadotrophins (Menopur, Gonal F, Puregon, Metrodin) combined with spontaneous sexual intercourse (without intrauterine insemination)

Treatment with hormonal stimulation using pills (Clomid, Nolvadex, Arimidex, Aromasin, Femara, etc.) combined with intrauterine insemination (IUI)

Treatment with hormonal stimulation using injections of gonadotrophins (Menopur, Gonal F, Puregon, Metrodin) combined with intrauterine insemination (IUI)

Treatment with intrauterine insemination without hormonal stimulation

Treatment with in vitro fertilization (IVF) or intra cytoplasmic sperm injection (ICSI)

Hormonal treatments

Please enter the number of months of hormonal treatment for your chronic pelvic pain during your life up until the day you completed this survey. Please enter '0' if you did not receive any hormonal treatment.

66. Please enter the number of months you have been on any of these hormonal treatments for your pelvic pain. If you have not been on any of these please enter '0'

GnRH agonists and/or antagonists (e.g. Zoladex, Synarel)

Danazol (e.g. Danol, Danocrine)

Progestins / progestogens (e.g. Provera, Primolut N, Visanne)

Contraceptive pills (e.g. Levlen, Noromin, Yasmin, Diane, Microgynon)

Sirena coil or Mirena

Aromatase inhibitors (e.g. Femara)

Health service usage in the past 2 months

These questions are all around the health/medical services you have used in the past two months only. Please don't include any health service usage that didn't occur in the two months prior to you filling in this survey.

67. Please record the dates of each physician visit in the last two months because of endometriosis, chronic pelvic pain or infertility. This relates to a visit to a general practitioner (GP), gynaecologist, surgeon, internal medicine specialist, psychiatrist or other specialist physicians (SP). Please specify an estimate of any transportation costs for each visit

Date of Visit 1	
Type of Visit 1 (e.g. GP, Gynaecologist, fertility specialist)	
Transportation cost Visit 1	
Date of Visit 2	
Type of Visit 2 (e.g. GP, Gynaecologist, fertility specialist)	
Transportation cost Visit 2	
Date of Visit 3	
Type of Visit 3 (e.g. GP, Gynaecologist, fertility specialist)	
Transportation cost Visit 3	
Date of Visit 4	
Type of Visit 4 (e.g. GP, Gynaecologist, fertility specialist)	
Transportation cost Visit 4	

68. Please enter all medication (including over the counter medication like ibuprofen) you have taken in the **last two months** related to endometriosis,pelvic pain or infertility. Please provide details of daily dose and number of days of medication use.

Brand name of medication 1	
Dosage of medication 1	
Number of days of medication 1	
Brand name of medication 2	
Dosage of medication 2	
Number of days of medication 2	
Brand name of medication 3	
Dosage of medication 3	
Number of days of medication 3	
Brand name of medication 4	
Dosage of medication 4	
Number of days of medication 4	
Brand name of medication 5	
Dosage of medication 5	
Number of days of medication 5	
Brand name of medication 6	
Dosage of medication 6	
Number of days of medication 6	

Monitoring tests

Please enter all laboratory and monitoring tests that you have undergone in the last two months because of endometriosis, pelvic pain or infertility.

69. Which of the following procedures have you had in the past two months?

	Number of procedures
Ultrasound scan (transvaginal, abdominal, transrectal)	
Ultrasound scan (kidney)	
Magnetic resonance imaging	
Computed tomography (CT scan)	
Intravenous pyelography (IVP)	
Barium enema	
Sigmoidoscopy	
Blood tests	
Bacteriology / culture	

Other (please specify type and number of procedures)

Hospitalisations

Hospitalisations (anything more than a simple out-patient visit) Please include all contacts with a hospital in the last two months where you had to lie in a bed or bed-like reclining chair, where you had a surgical procedure, and where you had egg retrieval and embryo transfer for IVF, even if done without an overnight stay)

70. How many times have you been in hospital in the last two months?

71. If you have been in hospital, can you provide a total for the amount of money all the hospital stays have cost you (i.e. how much you have paid out of your own pocket). Please include transportation costs as well.

72. If you were in hospital, what were the starting and ending dates for your first hospital visit ?

	DD		MM		YYYY
From Date (when you went into hospital)		/		/	
End Date (when you left hospital)		/		/	

73. If you were in hospital, what were the starting and ending dates for your second hospital visit ?

	DD		MM		YYYY
From Date (when you went into hospital)		/		/	
End Date (when you left hospital)		/		/	

74. If you were in hospital, what were the starting and ending dates for your third hospital visit ?

	DD		MM		YYYY
From Date (when you went into hospital)		/		/	
End Date (when you left hospital)		/		/	

75. If you were in hospital, what were the starting and ending dates for your fourth hospital visit ?

	DD		MM		YYYY
From Date (when you went into hospital)		/		/	
End Date (when you left hospital)		/		/	

Other treatments

Please enter all other treatments in the last two months related to endometriosis, pelvic pain or infertility. This also includes alternative/complementary treatments (e.g. homeopathy, acupuncture, psychologist, reflexology, etc.). Please enter all types of the SAME treatment under the one heading, so if you have seen three different homeopaths please enter it all under the therapy named "Homeopathy".

76. Other treatments 1

Type/name of therapy (e.g nutritionist) [ ]

Number of treatment sessions [ ]

Total cost (including transportation) [ ]

77. Other treatments 2

Type/name of therapy (e.g nutritionist) [ ]

Number of treatment sessions [ ]

Total cost (including transportation) [ ]

78. Other treatments 3

Type/name of therapy (e.g nutritionist) [ ]

Number of treatment sessions [ ]

Total cost (including transportation) [ ]

79. Other treatments 4

Type/name of therapy (e.g nutritionist) [ ]

Number of treatment sessions [ ]

Total cost (including transportation) [ ]

80. Other treatments 5

Type/name of therapy (e.g nutritionist)

Number of treatment sessions

Total cost (including transportation)

Additional support and informal care

Please specify the dates on which you received additional support with household activities (e.g. cleaning, ironing, child care) in the last two months because of endometriosis, pelvic pain and infertility. This covers support with household activities provided by professionals and provided by family, friends, neighbours etc. Provide a description of the type of support that your household received. Indicate the number of hours and out of pocket expenses for support received.

81. Additional support 1

Type of support

Number of hours of support

Total out of pocket cost

82. Additional support 2

Type of support

Number of hours of support

Total out of pocket cost

83. Additional support 3

Type of support

Number of hours of support

Total out of pocket cost

84. Additional support 4

Type of support

Number of hours of support

Total out of pocket cost

The impact of chronic pelvic pain on work, education and personal relationships

* 85. Have you ever lost time to education due to your chronic pelvic pain?

- ◯ Yes
- ◯ No

The impact of chronic pelvic pain on work, education and personal relationships

86. How did it effect your education (please select all that apply) ?

- [ ] Gave up my studies
- [ ] Changed studies (i.e. to a different course)
- [ ] Delayed my exam(s) or postponed assignments

Other (please explain)

87. Average number of days lost per month when you were studying due to your pelvic pain

88. Has chronic pelvic pain affected your job ?

- Yes
- No
- N/A - I'm not/haven't been employed in the last 12 months

89. If yes, how did it affect your job (please select all that apply)?

- [ ] Lost job (resigned/fired)
- [ ] Changed job
- [ ] Reduced work hours
- [ ] Other (please specify)

90. Average number of days per month you had to take off work due to your chronic pelvic pain ?

91. Have you been scared to tell your employer that you have chronic pelvic pain because you feared that it might affect your prospects?

- Yes
- No

92. Has chronic pelvic pain ever affected your personal relationships in a negative way?

- ○ Yes
- ○ No

93. If yes, how did it affect your personal relationships? (please select all that apply)

- ☐ Caused significant problems with your partner
- ☐ Created problems with your family
- ☐ Caused a relationship to split
- ☐ Made it difficult to look after children
- ☐ Affected friendships

If you would like to add more detail please do so here

Productivity

The following questions ask about the effect of endometriosis, pelvic pain and infertility on your ability to work and perform regular activities.

* 94. Are you currently employed (working for pay) ?

- ◯ Yes
- ◯ No

Productivity at work

The following questions are about the previous 7 days (not including today)

95. During the past **seven** days, how many hours did you miss from work because of problems associated with endometriosis, pelvic pain and infertility? Include hours you missed on sick days, times you went in late, left early, etc., because of endometriosis, pelvic pain and infertility. Do not include time you missed to participate in this survey.

[ ]

96. During the past **seven** days, how many hours did you miss from work because of any other reason, such as vacation or public holidays?

[ ]

97. During the past **seven** days, how many hours did you actually work?

[ ]

98. During the past **seven** days, how much did your pelvic pain and/or infertility affect your productivity while you were working?

Think about days you were limited in the amount or kind of work you could do, days you accomplished less than you would like, or days you could not do your work as carefully as usual. If pelvic pain and infertility affected your work only a little, choose a low number. Choose a high number if endometriosis, pelvic pain and infertility affected your work a great deal.

0 — 0 means that there was no effect on your work while 10 means that you were completely prevented from working — 10

[ ]

99. During the past **seven** days, how much did endometriosis, pelvic pain and infertility affect your ability to do your regular daily activities, other than work at a job?

By regular activities, we mean the usual activities you do, such as work around the house, shopping, childcare, exercising, studying, etc. Think about times you were limited in the amount or kind of activities you could do and times you accomplished less than you would like. If pelvic pain and infertility affected your activities only a little, choose a low number. Choose a high number if endometriosis, pelvic pain and infertility affected your activities a great deal.

0 means that there was no effect on your activities while 10 means that you were completely prevented from doing your usual activities

0 — 10

Thank you!

Thank you for participating in our survey, we really appreciate your time. We are planning to run some focus groups in Sydney in the latter half of 2017 on the impact of chronic pelvic pain. If you live in Sydney and would be interested in participating you can leave your contact details below. These will NOT be linked to your survey responses. Please read the information sheet here on what participating in the focus groups entails prior to leaving your details. Your involvement is completely voluntary and you may withdraw your interest at any time.

100. I am interested in participating in a focus group on chronic pelvic pain, please contact me with further information

Name ____________________

ZIP/Postal Code ____________

Email Address ____________________

Phone Number ____________________

101. We are also trying to make sure we include a variety of women with different experiences in our focus groups. To help us achieve this, please indicate how significantly impacted your life is due to your pelvic pain

- ◯ Very significant impact - It negatively affects my life on an almost daily or weekly basis
- ◯ Moderate impact - It negatively affects my life at least monthly
- ◯ Minor impact - It negatively affects my life occasionally
- ◯ No real impact - I don't really think my life is negatively affected by my pelvic pain
