# Supplementary material for: The cost of illness and economic burden of endometriosis and chronic pelvic pain in Australia: A national online survey
Source: PLoS One. 2019 Oct 10;14(10):e0223316. doi: 10.1371/journal.pone.0223316 (PMC6786587; doi:10.1371/journal.pone.0223316)
Supplement: S2 File — (PDF) [file pone.0223316.s002.pdf]

Survey:

The WERF EndoCost tool was developed by the World Endometriosis Research Foundation (WERF) EndoCost Consortium, and the original protocol consists of validated prospective hospital questionnaires and both retrospective and prospective patient questionnaires (1). Our study used the retrospective patient questionnaire component of the WERF EndoCost tool that was modified to an Australian demographic and healthcare context and hosted on SurveyMonkey (www.surveymonkey.com). The tool consisted of ninety-nine questions including direct healthcare costs (e.g. costs of medications and doctors visits), direct non-healthcare costs (e.g. transportation costs), and indirect costs of productivity loss. Total time to complete the survey was between 30-45 minutes. Modifications were made to income and ethnicity to adapt to Australian norms as per the Australian Bureau of Statistics (2). Brand names for pharmaceuticals were modified to reflect their Australian brand names. Analysis on other components of the WERF EndoCost tool (such as time to diagnosis, pelvic pain scores etc.) will be published separately.

The survey link was distributed via the social media platforms (Facebook, Twitter and Instagram) of Endometriosis Australia, EndoActive and Pelvic Pain Foundation of Australia. The total combined reach of these organisations on social media was just over 35k followers. Each organisation made two social media posts regarding the survey, the second post 3-5 weeks after the first. The survey link was active from February 2017 to April 2017, for a total of eight weeks. Data collection was closed once there had been no new responses for five days. Ethical approved was provided by the Western Sydney University Human Research Ethics Committee, approval number H12019.

Women were eligible to participate in the survey if they were aged 18-45, currently living in Australia and either had a surgically confirmed diagnosis of endometriosis, or if they had chronic pelvic pain from any cause. Chronic pelvic pain was defined as pain in the pelvis for at least six months that caused the woman to seek medical attention and if they had either; a

laparoscopy that did not show evidence of endometriosis or had not undergone a laparoscopy at the time of survey. All data collected was from participants themselves. Following standard practice in cost-of-illness studies, this study measured costs rather than test a specific hypothesis and so no sample size calculation was necessary (1).

Health care context:

In Australia, is there is a mix public and private health care system. All residents have automatic access to the public system comprising: (i) primary (general practitioners (GP), allied health and pharmaceuticals), and (ii) secondary care (hospital, in-patient and out-patient). The government provides subsided care, and so includes a copayment mechanism resulting in patient out-of-pocket costs. Taking private insurance does not preclude using public hospitals. The assumption in this study was that patients visited public providers.

Analysis:

A 'societal perspective' was adopted, incorporating (i) health sector impacts, often termed 'direct costs', (ii) productivity impacts, often termed 'indirect costs', and (iii) household costs (out-of-pocket costs, and in-kind carer time costs). This was a prevalence study and estimated costs regardless of time of diagnosis, if known. The immediate aim was to estimate average per person costs, and separately, for (i) women with a diagnosis of endometriosis, and (ii) women reported suffering from general chronic pelvic pain, without an associated diagnosis. Results from the sample were then extrapolated to the Australian female population, using secondary studies on prevalence of endometriosis and overall chronic pelvic pain.

The survey asks for recall from one or two months for most items, except fertility related treatments where there was lifetime recall. This was then extrapolated to estimated costs over one year, using techniques specific to the type of cost considered, outlined as follows:

Costs were estimated for AUD $ in 2017 prices. Following standard practice, to enable comparison of the economic burden between countries, costs were converted to International dollars (Int $) using purchasing power parity (PPP) conversion factors so that Int $1 is equivalent to US $1 in the United States (3).

Direct costs

Primary care costs

Cost included were pharmaceuticals, consultations with GP and allied health, specialist visits including allied health and complementary therapies, e.g. naturopathy, acupuncture, psychologist. Survey respondents noted pharmaceutical and dosage or services and number of sessions. Unit costs for pharmaceuticals are provided from the Pharmaceutical Benefits Scheme (PBS)(4). The manufacturer price minus the patient contribution is included as primary care cost. Unit costs for consultations and specialists are provided from Medicare Benefit Schedule (MBS) (5), again this is disaggregated into the proportion paid by the Government and that by the patient. To extrapolate to annual costs, it was assumed that costs incurred would repeat over the course of the year, and so the two-month cost was multiplied by 6 at the patient level (1).

Secondary care costs

This encompasses all hospital in-patient and out-patient care, including diagnostics and procedures, specialists, 'hotelier' costs associated with overnight stays, and the reported length of stay in hospital. To extrapolate to annual costs different assumptions were used conditional upon the type of cost incurred. For women, who have incurred hospital stays, it was assumed that the diagnostics and procedures reported over the two-month survey period would not be repeated continuously throughout the year (e.g. pelvic ultrasound and

laparoscopic removal of endometriosis), and so estimates for the two-month period were not extrapolated further. For women who had not yet incurred secondary costs, an estimate was made of the likely costs that would be incurred for the remainder of the year equivalent to the average two-month costs of those women who had incurred secondary costs. There is likely to be between-patient heterogeneity, however the main aim of the analysis was to estimate average costs over the population rather than per patient discrimination.

Out-of-pocket health care costs

This is the cost of primary and secondary care not subsidized by the PBS and MBS. Regarding pharmaceuticals, the patient pays a non-concessional $39.5 for each prescription. This was applied to each patient reporting use of pharmaceuticals. For repeat prescriptions, there is an annual out of pocket fee limit of $1,521.8, thereafter the cost per prescription reduces to the concessional rate ($6.40). Also included were patient transport and other costs associated with visits to primary and secondary providers. To extrapolate to annual costs, the two-month estimate was then multiplied by six.

Indirect costs: productivity impacts

Initial impact

A 'human capital approach' was taken where lost working time was valued by lost income to the person. The survey recorded weekly earning wage category which included the categories of: < $500, $501 to $1500, $1501 to $3000, $3001 to $4500, and > $4500 per week. The midpoint was taken in all categories, and an assumption that reported incomes over >$4,500 was equal to $5,000. Costs were divided into 'Absenteeism' and 'Presenteeism'. The former is measured by days absent from work, which was then multiplied by the pro rata daily income rate. The later was measured by the question 'During the past seven days, how much did your pelvic pain and/or infertility affect your productivity?' Responses from 0-10 were converted into associated percentages (e.g. 5 = 50%) and multiplied by the person's daily income rate.

To extrapolate to annual costs the two-month absenteeism and presenteeism costs were multiplied by six.

Multiplier impact

Reduced productivity has knock-on impacts to the wider economy, beyond individuals and/or companies affected, for example reduced household spending affects other business, and so forth. This knock-on effect is called the multiplier impact, and assumed to be 1.7 for absenteeism, and 1.97 for presenteeism using recent estimates and recommendations that all COI should incorporate multiplier effects (6). These multipliers were then applied to individual level survey responses to then estimate the full productivity impacts in the economy overall.

Indirect costs: carer impacts

Finally, the impact on carers was estimated, where the survey asked women if carers were required, time spent providing care and if there was a paid element. The analysis then divided carer costs into (i) 'professional', the paid element and (ii) 'in-kind', the unpaid element, where carer time was valued by the national average income level of $1,605/week and adjusted pro-rata. This was used to value the opportunity cost to volunteer carers.

Outcomes: Average costs per patient

The average cost per person was estimated across the categories described above and collated into an average for the sample as a whole, and also divided into the age categories of 18-24,25-30,31-38, 39+. Analysis was conducted by endometriosis and chronic pelvic pain separately. Mean and 95% confidence intervals were reported derived using bootstrapping with 5,000 replications(7).

Outcomes: Variation by pain severity

An *a priori* decision was made to explore whether average costs differ by pain severity. The survey asked women to rate pain from 1-10, and then was collapsed into four categories: 'Minimal' (1-2), 'Mild' (3-5), 'Moderate' (6-8), 'Severe' (9-10). Women were then stratified, and the costing analysis as described above was repeated.

Outcomes: Extrapolation to Australian population

Results from the survey were extrapolated by multiplying: (i) estimates of the prevalence of endometriosis using a rate of 10% (8), the most commonly accepted estimate (ii) the number of women in each age category (iii) average costs by age-category.

Sensitivity analysis

An 'analysis of extremes' was conducted, where the key structural assumptions of the main analysis were altered to generate lower bound estimates. First, the Human Capital Approach was substituted with the Frictional Cost Approach where productivity impacts were capped at 3 months with the assumption of replacement in the workforce. Second, to account for potential uncertainty regarding unit costs estimates all estimates of costs and productivity estimates were lowered by 10%. Third, the population prevalence of endometriosis and CPP was then lowered to 5%.

1. Simoens S, Hummelshoj L, Dunselman G, Brandes I, Dirksen C, D'Hooghe T, et al. Endometriosis cost assessment (the EndoCost study): a cost-of-illness study protocol. Gynecol Obstet Invest. 2011;71(3):170-6.
2. Australian Bureau of Statistics. 1249.0 - Australian Standard Classification of Cultural and Ethnic Groups (ASCCEG) 2016 [Available from: http://www.abs.gov.au/ausstats/abs@.nsf/mf/1249.0.
3. The World Bank. PPP conversion factor, GDP (LCU per international $) 2015 [Available from: http://data.worldbank.org/indicator/PA.NUS.PPP.
4. Australian Government Department of Health. The Pharmaceutical Benefits Scheme (PBS) 2018 [Available from: http://www.pbs.gov.au/browse/medicine-listing.
5. Australian Government Department of Health. Medicare Benefits Schedule (MBS) 2018 [Available from: http://www.mbsonline.gov.au/internet/mbsonline/publishing.nsf/Content/news-2018-07-01-latest-news-July.
6. Stromberg C, Aboagye E, Hagberg J, Bergstrom G, Lohela-Karlsson M. Estimating the Effect and Economic Impact of Absenteeism, Presenteeism, and Work Environment-Related Problems on Reductions in Productivity from a Managerial Perspective. Value Health. 2017;20(8):1058-64.
7. Rascati KL, Smith MJ, Neilands T. Dealing with skewed data: an example using asthma-related costs of medicaid clients. Clin Ther. 2001;23(3):481-98.
8. Vigano P, Parazzini F, Somigliana E, Vercellini P. Endometriosis: epidemiology and aetiological factors. Best Pract Res Clin Obstet Gynaecol. 2004;18(2):177-200.
