# Supplementary material for: The cost of illness and economic burden of endometriosis and chronic pelvic pain in Australia: A national online survey
Source: PLoS One. 2019 Oct 10;14(10):e0223316. doi: 10.1371/journal.pone.0223316 (PMC6786587; doi:10.1371/journal.pone.0223316)
Supplement: S4 File — (DOCX) [file pone.0223316.s004.docx]

Companion information:

| **Variable Information** | | | | | | | | |
| --- | --- | --- | --- | --- | --- | --- | --- | --- |
| Variable | Position | Label | Measurement Level | Role | Column Width | Alignment | Print Format | Write Format |
| q0003 | 2 | Highest level of education: | Nominal | Input | 8 | Right | F8.2 | F8.2 |
| q0004 | 3 | Current relationship status: | Nominal | Input | 8 | Right | F8.2 | F8.2 |
| q0005_0001 | 4 | Occupation: (please tick all that apply) | Nominal | Input | 8 | Right | F8.2 | F8.2 |
| q0005_0002 | 5 | Occupation: (please tick all that apply) | Nominal | Input | 8 | Right | F8.2 | F8.2 |
| q0005_0003 | 6 | Occupation: (please tick all that apply) | Nominal | Input | 8 | Right | F8.2 | F8.2 |
| q0005_0004 | 7 | Occupation: (please tick all that apply) | Nominal | Input | 8 | Right | F8.2 | F8.2 |
| q0005_0005 | 8 | Occupation: (please tick all that apply) | Nominal | Input | 8 | Right | F8.2 | F8.2 |
| q0005_0006 | 9 | Occupation: (please tick all that apply) | Nominal | Input | 8 | Right | F8.2 | F8.2 |
| q0005_0007 | 10 | Occupation: (please tick all that apply) | Nominal | Input | 8 | Right | F8.2 | F8.2 |
| q0005_0008 | 11 | Occupation: (please tick all that apply) | Nominal | Input | 8 | Right | F8.2 | F8.2 |
| q0005_other | 12 | Other (please specify) | Nominal | Input | 50 | Left | A70 | A70 |
| q0006 | 13 | Which of the following best describes your ethnicity: | Nominal | Input | 8 | Right | F8.2 | F8.2 |
| q0006_other | 14 | Other (please specify) | Nominal | Input | 50 | Left | A31 | A31 |
| q0007 | 15 | Do you have children ? | Nominal | Input | 8 | Right | F8.2 | F8.2 |
| q0008 | 16 | Income level - What is your average weekly earnings before tax | Nominal | Input | 8 | Right | F8.2 | F8.2 |
| q0009 | 17 | What is the cause of your chronic pelvic pain? | Nominal | Input | 8 | Right | F8.2 | F8.2 |
| q0010 | 18 | What year did you first experience symptoms of chronic pelvic pain? | Nominal | Input | 50 | Left | A149 | A149 |
| q0012 | 19 | Year you first sought medical help for any of the above symptoms in Q11 | Nominal | Input | 50 | Left | A125 | A125 |
| q0013 | 20 | What year did you get diagnosed with endometriosis ? | Nominal | Input | 50 | Left | A36 | A36 |
| q0014 | 21 | How many doctors did you see before you were diagnosed with endometriosis? | Nominal | Input | 50 | Left | A172 | A172 |
| q0015 | 22 | How many complementary therapists (e.g. acupuncturists, homeopaths, naturopaths) did you see before you were diagnosed with endometriosis? | Nominal | Input | 50 | Left | A52 | A52 |
| q0016 | 23 | What stage was your endometriosis after your most recent laparoscopy (Stage I-IV) | Nominal | Input | 8 | Right | F8.2 | F8.2 |
| q0017 | 24 | How many doctors have you seen to get a diagnosis for your chronic pelvic pain ? | Nominal | Input | 50 | Left | A93 | A93 |
| q0018 | 25 | If you have received a diagnosis for the cause of your chronic pelvic pain for your doctor, please write it below | Nominal | Input | 50 | Left | A113 | A113 |
| q0019 | 26 | How many complementary therapists have you seen for your chronic pelvic pain (e.g. acupuncturist, homeopath) ? | Nominal | Input | 50 | Left | A74 | A74 |
| q0020 | 27 | In the last 3 months, have you had pelvic pain with your periods? | Nominal | Input | 8 | Right | F8.2 | F8.2 |
| q0021 | 28 | How often have you had pelvic pain with your periods in the last 3 months? | Nominal | Input | 8 | Right | F8.2 | F8.2 |
| q0022 | 29 | In the last 3 months, have you taken pain-killers for the pain that are prescribed for you by a doctor? | Nominal | Input | 8 | Right | F8.2 | F8.2 |
| q0023 | 30 | In the last 3 months, have you taken pain-killers for the pain, bought over the counter without prescription? | Nominal | Input | 8 | Right | F8.2 | F8.2 |
| q0024 | 31 | In the last 3 months, has your period pain prevented you from going to work or carrying out your daily activities (even if taking pain-killers) ? | Nominal | Input | 8 | Right | F8.2 | F8.2 |
| q0025 | 32 | In the last 3 months, have you had to lie down for any part of the day or longer because of your period pain? | Nominal | Input | 8 | Right | F8.2 | F8.2 |
| q0026 | 33 | Please circle on the following scale, going from no pain (0) to worst possible pain (10), the number that indicates how severe your period pain has been on average in the last 3 months: | Nominal | Input | 50 | Left | A2 | A2 |
| q0027 | 34 | Please circle on the following scale, going from no pain (0) to worst possible pain (10), the number that indicates how severe your period pain has been at its worst in the last 3 months: | Nominal | Input | 50 | Left | A2 | A2 |
| q0045 | 35 | Do you take pain-killers for this pain, prescribed for you by a doctor? | Nominal | Input | 8 | Right | F8.2 | F8.2 |
| q0046 | 36 | Do you take pain-killers for this pain that you can buy without a prescription? (e.g. Aspirin, Nurofen, Paracetamol) | Nominal | Input | 8 | Right | F8.2 | F8.2 |
| SF1 | 37 | SF1 | Nominal | Input | 8 | Right | F8.2 | F8.2 |
| SF2 | 38 | SF2 | Nominal | Input | 8 | Right | F8.2 | F8.2 |
| SF3a | 39 | SF3a | Scale | Input | 8 | Right | F8.2 | F8.2 |
| SF3b | 40 | SF3b | Scale | Input | 8 | Right | F8.2 | F8.2 |
| SF3c | 41 | SF3c | Scale | Input | 8 | Right | F8.2 | F8.2 |
| SF3d | 42 | SF3d | Scale | Input | 8 | Right | F8.2 | F8.2 |
| SF3e | 43 | SF3e | Scale | Input | 8 | Right | F8.2 | F8.2 |
| SF3f | 44 | SF3f | Scale | Input | 8 | Right | F8.2 | F8.2 |
| SF3g | 45 | SF3g | Scale | Input | 8 | Right | F8.2 | F8.2 |
| SF3h | 46 | SF3h | Scale | Input | 8 | Right | F8.2 | F8.2 |
| SF3i | 47 | SF3i | Scale | Input | 8 | Right | F8.2 | F8.2 |
| SF3j | 48 | SF3j | Scale | Input | 8 | Right | F8.2 | F8.2 |
| SF4a | 49 | SF4a | Scale | Input | 8 | Right | F8.2 | F8.2 |
| SF4b | 50 | SF4b | Scale | Input | 8 | Right | F8.2 | F8.2 |
| SF4c | 51 | SF4c | Scale | Input | 8 | Right | F8.2 | F8.2 |
| SF4d | 52 | SF4d | Scale | Input | 8 | Right | F8.2 | F8.2 |
| SF5a | 53 | SF5a | Scale | Input | 8 | Right | F8.2 | F8.2 |
| SF5b | 54 | SF5b | Scale | Input | 8 | Right | F8.2 | F8.2 |
| SF5c | 55 | SF5c | Scale | Input | 8 | Right | F8.2 | F8.2 |
| SF6 | 56 | SF6 | Nominal | Input | 8 | Right | F8.2 | F8.2 |
| SF7 | 57 | SF7 | Nominal | Input | 8 | Right | F8.2 | F8.2 |
| SF8 | 58 | SF8 | Nominal | Input | 8 | Right | F8.2 | F8.2 |
| SF9a | 59 | SF9a | Scale | Input | 8 | Right | F8.2 | F8.2 |
| SF9b | 60 | SF9b | Scale | Input | 8 | Right | F8.2 | F8.2 |
| SF9c | 61 | SF9c | Scale | Input | 8 | Right | F8.2 | F8.2 |
| SF9d | 62 | SF9d | Scale | Input | 8 | Right | F8.2 | F8.2 |
| SF9e | 63 | SF9e | Scale | Input | 8 | Right | F8.2 | F8.2 |
| SF9f | 64 | SF9f | Scale | Input | 8 | Right | F8.2 | F8.2 |
| SF9g | 65 | SF9g | Scale | Input | 8 | Right | F8.2 | F8.2 |
| SF9h | 66 | SF9h | Scale | Input | 8 | Right | F8.2 | F8.2 |
| SF9i | 67 | SF9i | Scale | Input | 8 | Right | F8.2 | F8.2 |
| SF10 | 68 | SF10 | Nominal | Input | 8 | Right | F8.2 | F8.2 |
| SF11a | 69 | SF11a | Scale | Input | 8 | Right | F8.2 | F8.2 |
| SF11b | 70 | SF11b | Scale | Input | 8 | Right | F8.2 | F8.2 |
| SF11c | 71 | SF11c | Scale | Input | 8 | Right | F8.2 | F8.2 |
| SF11d | 72 | SF11d | Scale | Input | 8 | Right | F8.2 | F8.2 |
| q0063_0001 | 73 | Laparoscopy (key hole surgery) | Nominal | Input | 8 | Right | F8.2 | F8.2 |
| q0063_0002 | 74 | Laparotomy (open surgery) | Nominal | Input | 8 | Right | F8.2 | F8.2 |
| q0064_0001 | 75 | Diagnosis and/or removal of endometriosis | Nominal | Input | 8 | Right | F8.2 | F8.2 |
| q0064_0002 | 76 | Removal of one ovary or both ovaries (via keyhole surgery) | Nominal | Input | 8 | Right | F8.2 | F8.2 |
| q0064_0003 | 77 | Removal of one ovary or both ovaries (via open surgery) | Nominal | Input | 8 | Right | F8.2 | F8.2 |
| q0064_0004 | 78 | Removal of uterus/womb (via key hole surgery) | Nominal | Input | 8 | Right | F8.2 | F8.2 |
| q0064_0005 | 79 | Removal of uterus/womb (via open surgery) | Nominal | Input | 8 | Right | F8.2 | F8.2 |
| q0064_0006 | 80 | Removal of uterus/womb and ovaries in the same procedure (via key hole surgery) | Nominal | Input | 8 | Right | F8.2 | F8.2 |
| q0064_0007 | 81 | Removal of uterus/womb and ovaries in the same procedure (via open surgery) | Nominal | Input | 8 | Right | F8.2 | F8.2 |
| q0065_0001 | 82 | Treatment with hormonal stimulation using pills (Clomid, Nolvadex, Arimidex, Aromasin, Femara, etc.) combined with spontaneous sexual intercourse (without intrauterine insemination) | Nominal | Input | 8 | Right | F8.2 | F8.2 |
| q0065_0002 | 83 | Treatment with hormonal stimulation using injections of gonadotrophins (Menopur, Gonal F, Puregon, Metrodin) combined with spontaneous sexual intercourse (without intrauterine insemination) | Nominal | Input | 8 | Right | F8.2 | F8.2 |
| q0065_0003 | 84 | Treatment with hormonal stimulation using pills (Clomid, Nolvadex, Arimidex, Aromasin, Femara, etc.) combined with intrauterine insemination (IUI) | Nominal | Input | 8 | Right | F8.2 | F8.2 |
| q0065_0004 | 85 | Treatment with hormonal stimulation using injections of gonadotrophins (Menopur, Gonal F, Puregon, Metrodin) combined with intrauterine insemination (IUI) | Nominal | Input | 8 | Right | F8.2 | F8.2 |
| q0065_0005 | 86 | Treatment with intrauterine insemination without hormonal stimulation | Nominal | Input | 8 | Right | F8.2 | F8.2 |
| q0065_0006 | 87 | Treatment with in vitro fertilization (IVF) or intra cytoplasmic sperm injection (ICSI) | Nominal | Input | 8 | Right | F8.2 | F8.2 |
| q0066_0001 | 88 | GnRH agonists and/or antagonists (e.g. Zoladex, Synarel) | Nominal | Input | 8 | Right | F8.2 | F8.2 |
| q0066_0002 | 89 | Danazol (e.g. Danol, Danocrine) | Nominal | Input | 8 | Right | F8.2 | F8.2 |
| q0066_0003 | 90 | Progestins / progestogens (e.g. Provera, Primolut N, Visanne) | Nominal | Input | 8 | Right | F8.2 | F8.2 |
| q0066_0004 | 91 | Contraceptive pills (e.g. Levlen, Noromin, Yasmin, Diane, Microgynon) | Nominal | Input | 8 | Right | F8.2 | F8.2 |
| q0066_0005 | 92 | Sirena coil or Mirena | Nominal | Input | 8 | Right | F8.2 | F8.2 |
| q0066_0006 | 93 | Aromatase inhibitors (e.g. Femara) | Nominal | Input | 8 | Right | F8.2 | F8.2 |
| q0067_0002 | 95 | Type of Visit 1 (e.g. GP, Gynaecologist, fertility specialist) | Nominal | Input | 50 | Left | A48 | A48 |
| q0067_0003 | 96 | Transportation cost Visit 1 | Nominal | Input | 50 | Left | A42 | A42 |
| q0067_0005 | 98 | Type of Visit 2 (e.g. GP, Gynaecologist, fertility specialist) | Nominal | Input | 50 | Left | A34 | A34 |
| q0067_0006 | 99 | Transportation cost Visit 2 | Nominal | Input | 50 | Left | A40 | A40 |
| q0067_0008 | 101 | Type of Visit 3 (e.g. GP, Gynaecologist, fertility specialist) | Nominal | Input | 50 | Left | A44 | A44 |
| q0067_0009 | 102 | Transportation cost Visit 3 | Nominal | Input | 50 | Left | A42 | A42 |
| q0067_0011 | 104 | Type of Visit 4 (e.g. GP, Gynaecologist, fertility specialist) | Nominal | Input | 50 | Left | A51 | A51 |
| q0067_0012 | 105 | Transportation cost Visit 4 | Nominal | Input | 50 | Left | A27 | A27 |
| q0067_0014 | 107 | Type of Visit 5 (e.g. GP, Gynaecologist, fertility specialist) | Nominal | Input | 50 | Left | A55 | A55 |
| q0067_0015 | 108 | Transportation cost Visit 5 | Nominal | Input | 50 | Left | A6 | A6 |
| q0067_0017 | 110 | Type of Visit 6 (e.g. GP, Gynaecologist, fertility specialist) | Nominal | Input | 50 | Left | A33 | A33 |
| q0067_0018 | 111 | Transportation cost Visit 6 | Nominal | Input | 50 | Left | A3 | A3 |
| q0068_0001 | 112 | Brand name of medication 1 | Nominal | Input | 50 | Left | A40 | A40 |
| q0068_0002 | 113 | Dosage of medication 1 | Nominal | Input | 50 | Left | A60 | A60 |
| q0068_0003 | 114 | Number of days of medication 1 | Nominal | Input | 50 | Left | A41 | A41 |
| q0068_0004 | 115 | Brand name of medication 2 | Nominal | Input | 50 | Left | A28 | A28 |
| q0068_0005 | 116 | Dosage of medication 2 | Nominal | Input | 50 | Left | A65 | A65 |
| q0068_0006 | 117 | Number of days of medication 2 | Nominal | Input | 50 | Left | A59 | A59 |
| q0068_0007 | 118 | Brand name of medication 3 | Nominal | Input | 50 | Left | A32 | A32 |
| q0068_0008 | 119 | Dosage of medication 3 | Nominal | Input | 50 | Left | A54 | A54 |
| q0068_0009 | 120 | Number of days of medication 3 | Nominal | Input | 50 | Left | A41 | A41 |
| q0068_0010 | 121 | Brand name of medication 4 | Nominal | Input | 50 | Left | A38 | A38 |
| q0068_0011 | 122 | Dosage of medication 4 | Nominal | Input | 50 | Left | A33 | A33 |
| q0068_0012 | 123 | Number of days of medication 4 | Nominal | Input | 50 | Left | A41 | A41 |
| q0068_0013 | 124 | Brand name of medication 5 | Nominal | Input | 50 | Left | A55 | A55 |
| q0068_0014 | 125 | Dosage of medication 5 | Nominal | Input | 50 | Left | A43 | A43 |
| q0068_0015 | 126 | Number of days of medication 5 | Nominal | Input | 50 | Left | A49 | A49 |
| q0068_0016 | 127 | Brand name of medication 6 | Nominal | Input | 50 | Left | A37 | A37 |
| q0068_0017 | 128 | Dosage of medication 6 | Nominal | Input | 50 | Left | A33 | A33 |
| q0068_0018 | 129 | Number of days of medication 6 | Nominal | Input | 50 | Left | A20 | A20 |
| q0069_0001_0001 | 130 | Ultrasound scan (transvaginal, abdominal, transrectal) - Number of procedures | Nominal | Input | 8 | Right | F8.2 | F8.2 |
| q0069_0002_0001 | 131 | Ultrasound scan (kidney) - Number of procedures | Nominal | Input | 8 | Right | F8.2 | F8.2 |
| q0069_0003_0001 | 132 | Magnetic resonance imaging - Number of procedures | Nominal | Input | 8 | Right | F8.2 | F8.2 |
| q0069_0004_0001 | 133 | Computed tomography (CT scan) - Number of procedures | Nominal | Input | 8 | Right | F8.2 | F8.2 |
| q0069_0005_0001 | 134 | Intravenous pyelography (IVP) - Number of procedures | Nominal | Input | 8 | Right | F8.2 | F8.2 |
| q0069_0006_0001 | 135 | Barium enema - Number of procedures | Nominal | Input | 8 | Right | F8.2 | F8.2 |
| q0069_0007_0001 | 136 | Sigmoidoscopy - Number of procedures | Nominal | Input | 8 | Right | F8.2 | F8.2 |
| q0069_0008_0001 | 137 | Blood tests - Number of procedures | Nominal | Input | 8 | Right | F8.2 | F8.2 |
| q0069_0009_0001 | 138 | Bacteriology / culture - Number of procedures | Nominal | Input | 8 | Right | F8.2 | F8.2 |
| q0069_other | 139 | Other (please specify type and number of procedures) | Nominal | Input | 50 | Left | A53 | A53 |
| q0070 | 140 | How many times have you been in hospital in the last two months? | Nominal | Input | 50 | Left | A2 | A2 |
| q0071 | 141 | If you have been in hospital, can you provide a total for the amount of money all the hospital stays have cost you (i.e. how much you have paid out of your own pocket). Please include transportation costs as well. | Nominal | Input | 50 | Left | A88 | A88 |
| q0076_0001 | 150 | Type/name of therapy (e.g nutritionist) | Nominal | Input | 50 | Left | A50 | A50 |
| q0076_0002 | 151 | Number of treatment sessions | Nominal | Input | 50 | Left | A27 | A27 |
| q0076_0003 | 152 | Total cost (including transportation) | Nominal | Input | 50 | Left | A54 | A54 |
| q0077_0001 | 153 | Type/name of therapy (e.g nutritionist) | Nominal | Input | 50 | Left | A83 | A83 |
| q0077_0002 | 154 | Number of treatment sessions | Nominal | Input | 50 | Left | A25 | A25 |
| q0077_0003 | 155 | Total cost (including transportation) | Nominal | Input | 50 | Left | A23 | A23 |
| q0078_0001 | 156 | Type/name of therapy (e.g nutritionist) | Nominal | Input | 50 | Left | A48 | A48 |
| q0078_0002 | 157 | Number of treatment sessions | Nominal | Input | 50 | Left | A15 | A15 |
| q0078_0003 | 158 | Total cost (including transportation) | Nominal | Input | 50 | Left | A38 | A38 |
| q0079_0001 | 159 | Type/name of therapy (e.g nutritionist) | Nominal | Input | 50 | Left | A13 | A13 |
| q0079_0002 | 160 | Number of treatment sessions | Nominal | Input | 50 | Left | A23 | A23 |
| q0079_0003 | 161 | Total cost (including transportation) | Nominal | Input | 50 | Left | A6 | A6 |
| q0080_0001 | 162 | Type/name of therapy (e.g nutritionist) | Nominal | Input | 50 | Left | A12 | A12 |
| q0080_0002 | 163 | Number of treatment sessions | Nominal | Input | 50 | Left | A1 | A1 |
| q0080_0003 | 164 | Total cost (including transportation) | Nominal | Input | 50 | Left | A4 | A4 |
| q0081_0001 | 165 | Type of support | Nominal | Input | 50 | Left | A78 | A78 |
| q0081_0002 | 166 | Number of hours of support | Nominal | Input | 50 | Left | A31 | A31 |
| q0081_0003 | 167 | Total out of pocket cost | Nominal | Input | 50 | Left | A25 | A25 |
| q0082_0001 | 168 | Type of support | Nominal | Input | 50 | Left | A53 | A53 |
| q0082_0002 | 169 | Number of hours of support | Nominal | Input | 50 | Left | A12 | A12 |
| q0082_0003 | 170 | Total out of pocket cost | Nominal | Input | 50 | Left | A24 | A24 |
| q0083_0001 | 171 | Type of support | Nominal | Input | 50 | Left | A51 | A51 |
| q0083_0002 | 172 | Number of hours of support | Nominal | Input | 50 | Left | A11 | A11 |
| q0083_0003 | 173 | Total out of pocket cost | Nominal | Input | 50 | Left | A4 | A4 |
| q0084_0001 | 174 | Type of support | Nominal | Input | 50 | Left | A49 | A49 |
| q0084_0002 | 175 | Number of hours of support | Nominal | Input | 50 | Left | A5 | A5 |
| q0084_0003 | 176 | Total out of pocket cost | Nominal | Input | 50 | Left | A4 | A4 |
| q0088 | 177 | Has chronic pelvic pain affected your job ? | Nominal | Input | 8 | Right | F8.2 | F8.2 |
| q0089_0001 | 178 | If yes, how did it affect your job (please select all that apply)? | Nominal | Input | 8 | Right | F8.2 | F8.2 |
| q0089_0002 | 179 | If yes, how did it affect your job (please select all that apply)? | Nominal | Input | 8 | Right | F8.2 | F8.2 |
| q0089_0003 | 180 | If yes, how did it affect your job (please select all that apply)? | Nominal | Input | 8 | Right | F8.2 | F8.2 |
| q0089_0004 | 181 | If yes, how did it affect your job (please select all that apply)? | Nominal | Input | 8 | Right | F8.2 | F8.2 |
| q0089_other | 182 | Other (please specify) | Nominal | Input | 50 | Left | A779 | A779 |
| q0090 | 183 | Average number of days per month you had to take off work due to your chronic pelvic pain ? | Nominal | Input | 50 | Left | A102 | A102 |
| q0091 | 184 | Have you been scared to tell your employer that you have chronic pelvic pain because you feared that it might affect your prospects? | Nominal | Input | 8 | Right | F8.2 | F8.2 |
| q0094 | 185 | Are you currently employed (working for pay) ? | Nominal | Input | 8 | Right | F8.2 | F8.2 |
| q0095 | 186 | During the past seven days, how many hours did you miss from work because of problems associated with endometriosis, pelvic pain and infertility? Include hours you missed on sick days, times you went in late, left early, etc., because of endometriosis, pe | Nominal | Input | 50 | Left | A2 | A2 |
| q0096 | 187 | During the past seven days, how many hours did you miss from work because of any other reason, such as vacation or public holidays? | Nominal | Input | 50 | Left | A53 | A53 |
| q0097 | 188 | During the past seven days, how many hours did you actually work? | Nominal | Input | 50 | Left | A49 | A49 |
| q0098 | 189 | During the past seven days, how much did your pelvic pain and/or infertility affect your productivity while you were working? Think about days you were limited in the amount or kind of work you could do, days you accomplished less than you would like, or | Nominal | Input | 50 | Left | A2 | A2 |
| q0099 | 190 | During the past seven days, how much did endometriosis, pelvic pain and infertility affect your ability to do your regular daily activities, other than work at a job?  By regular activities, we mean the usual activities you do, such as work around the hou | Nominal | Input | 50 | Left | A2 | A2 |
| Variables in the working file | | | | | | | | |

| **Variable Values** |  |  |
| --- | --- | --- |
| Value | Label |  |
| q0003 | 1.00 | Primary school |
|  | 2.00 | Lower secondary (Year 10) |
|  | 3.00 | Upper secondary (Year 12) |
|  | 4.00 | Post-secondary, not university (i.e. TAFE or vocational coll |
|  | 5.00 | University |
|  | 6.00 | Postgraduate |
| q0004 | 1.00 | Single |
|  | 2.00 | Married / De Facto (living with partner) |
|  | 3.00 | In a relationship but not living with partner. |
|  | 4.00 | Divorced / separated |
|  | 5.00 | Widowed |
| q0005_0001 | 1.00 | Employee |
| q0005_0002 | 1.00 | Self-employed |
| q0005_0003 | 1.00 | Home duties / caring for children or family |
| q0005_0004 | 1.00 | In education (going to school, university, etc.) |
| q0005_0005 | 1.00 | Doing voluntary work |
| q0005_0006 | 1.00 | Unable to work because of chronic pelvic pain symptoms |
| q0005_0007 | 1.00 | Unable to work for other reasons |
| q0005_0008 | 1.00 | Other (please specify) |
| q0006 | .00 | Other (please specify) |
|  | 1.00 | Caucasian |
|  | 2.00 | Asian |
|  | 3.00 | Aboriginal/TSI |
|  | 4.00 | Polynesian |
|  | 5.00 | Maori |
|  | 6.00 | Unknown |
| q0007 | 1.00 | Yes |
|  | 2.00 | No |
| q0008 | 1.00 | < $500 per week |
|  | 2.00 | $501 to $1500 per week |
|  | 3.00 | $1501 to $3000 per week |
|  | 4.00 | $3001 to $4500 per week |
|  | 5.00 | > $4500 per week |
| q0009 | 1.00 | Endometriosis (diagnosed via laparoscopy) |
|  | 2.00 | Any other cause (including suspected endometriosis, chronic |
| q0016 | 1.00 | Stage I (minimal) |
|  | 2.00 | Stage II (mild) |
|  | 3.00 | Stage III (moderate) |
|  | 4.00 | Stage IV (severe) |
|  | 5.00 | I can't remember |
| q0020 | 1.00 | Yes |
|  | 2.00 | No |
| q0021 | 1.00 | Occasionally (with 1 of my last 3 periods) |
|  | 2.00 | Often (with 2 in 3 of my last 3 periods) |
|  | 3.00 | Always (with all of my last 3 periods) |
| q0022 | 1.00 | No |
|  | 2.00 | Yes |
| q0023 | 1.00 | Yes |
|  | 2.00 | No |
| q0024 | 1.00 | Never |
|  | 2.00 | Occasionally (in 1 of my last 3 periods) |
|  | 3.00 | Often (in 2 of my last 3 periods) |
|  | 4.00 | Always (in all of my last 3 periods) |
| q0025 | 1.00 | Never |
|  | 2.00 | Occasionally  (in 1 of my last 3 periods) |
|  | 3.00 | Often (in 2 of my last 3 periods) |
|  | 4.00 | Always (in 3 of my last 3 periods) |
| q0045 | 1.00 | Yes |
|  | 2.00 | No |
| q0046 | 1.00 | Yes |
|  | 2.00 | No |
| q0050 | 1.00 | Excellent |
|  | 2.00 | Very good |
|  | 3.00 | Good |
|  | 4.00 | Fair |
|  | 5.00 | Poor |
| q0051 | 1.00 | Much better than one year ago |
|  | 2.00 | Somewhat better than one year ago |
|  | 3.00 | About the same |
|  | 4.00 | Somewhat worse now than one year ago |
|  | 5.00 | Much worse now than one year ago |
| q0052_0001 | 1.00 | Yes, Limited a lot |
|  | 2.00 | Yes, Limited a little |
|  | 3.00 | No, not limited at all |
| q0052_0002 | 1.00 | Yes, Limited a lot |
|  | 2.00 | Yes, Limited a little |
|  | 3.00 | No, not limited at all |
| q0052_0003 | 1.00 | Yes, Limited a lot |
|  | 2.00 | Yes, Limited a little |
|  | 3.00 | No, not limited at all |
| q0052_0004 | 1.00 | Yes, Limited a lot |
|  | 2.00 | Yes, Limited a little |
|  | 3.00 | No, not limited at all |
| q0052_0005 | 1.00 | Yes, Limited a lot |
|  | 2.00 | Yes, Limited a little |
|  | 3.00 | No, not limited at all |
| q0052_0006 | 1.00 | Yes, Limited a lot |
|  | 2.00 | Yes, Limited a little |
|  | 3.00 | No, not limited at all |
| q0052_0007 | 1.00 | Yes, Limited a lot |
|  | 2.00 | Yes, Limited a little |
|  | 3.00 | No, not limited at all |
| q0052_0008 | 1.00 | Yes, Limited a lot |
|  | 2.00 | Yes, Limited a little |
|  | 3.00 | No, not limited at all |
| q0052_0009 | 1.00 | Yes, Limited a lot |
|  | 2.00 | Yes, Limited a little |
|  | 3.00 | No, not limited at all |
| q0052_0010 | 1.00 | Yes, Limited a lot |
|  | 2.00 | Yes, Limited a little |
|  | 3.00 | No, not limited at all |
| q0053_0001 | 1.00 | All of the time |
|  | 2.00 | Most of the time |
|  | 3.00 | Some of the time |
|  | 4.00 | A little of the time |
|  | 5.00 | None of the time |
| q0053_0002 | 1.00 | All of the time |
|  | 2.00 | Most of the time |
|  | 3.00 | Some of the time |
|  | 4.00 | A little of the time |
|  | 5.00 | None of the time |
| q0053_0003 | 1.00 | All of the time |
|  | 2.00 | Most of the time |
|  | 3.00 | Some of the time |
|  | 4.00 | A little of the time |
|  | 5.00 | None of the time |
| q0053_0004 | 1.00 | All of the time |
|  | 2.00 | Most of the time |
|  | 3.00 | Some of the time |
|  | 4.00 | A little of the time |
|  | 5.00 | None of the time |
| q0054_0001 | 1.00 | All of the time |
|  | 2.00 | Most of the time |
|  | 3.00 | Some of the time |
|  | 4.00 | A little of the time |
|  | 5.00 | None of the time |
| q0054_0002 | 1.00 | All of the time |
|  | 2.00 | Most of the time |
|  | 3.00 | Some of the time |
|  | 4.00 | A little of the time |
|  | 5.00 | None of the time |
| q0054_0003 | 1.00 | All of the time |
|  | 2.00 | Most of the time |
|  | 3.00 | Some of the time |
|  | 4.00 | A little of the time |
|  | 5.00 | None of the time |
| q0055 | 1.00 | Not at all |
|  | 2.00 | Slightly |
|  | 3.00 | Moderately |
|  | 4.00 | Quite a bit |
|  | 5.00 | Extremely |
| q0056 | 1.00 | None |
|  | 2.00 | Very mild |
|  | 3.00 | Mild |
|  | 4.00 | Moderate |
|  | 5.00 | Severe |
|  | 6.00 | Very severe |
| q0057 | 1.00 | Not at all |
|  | 2.00 | Slightly |
|  | 3.00 | Moderately |
|  | 4.00 | Quite a bit |
|  | 5.00 | Extremely |
| q0058_0001 | 1.00 | All of the time |
|  | 2.00 | Most of the time |
|  | 3.00 | Some of the time |
|  | 4.00 | A little of the time |
|  | 5.00 | None of the time |
| q0058_0002 | 1.00 | All of the time |
|  | 2.00 | Most of the time |
|  | 3.00 | Some of the time |
|  | 4.00 | A little of the time |
|  | 5.00 | None of the time |
| q0058_0003 | 1.00 | All of the time |
|  | 2.00 | Most of the time |
|  | 3.00 | Some of the time |
|  | 4.00 | A little of the time |
|  | 5.00 | None of the time |
| q0058_0004 | 1.00 | All of the time |
|  | 2.00 | Most of the time |
|  | 3.00 | Some of the time |
|  | 4.00 | A little of the time |
|  | 5.00 | None of the time |
| q0058_0005 | 1.00 | All of the time |
|  | 2.00 | Most of the time |
|  | 3.00 | Some of the time |
|  | 4.00 | A little of the time |
|  | 5.00 | None of the time |
| q0058_0006 | 1.00 | All of the time |
|  | 2.00 | Most of the time |
|  | 3.00 | Some of the time |
|  | 4.00 | A little of the time |
|  | 5.00 | None of the time |
| q0058_0007 | 1.00 | All of the time |
|  | 2.00 | Most of the time |
|  | 3.00 | Some of the time |
|  | 4.00 | A little of the time |
|  | 5.00 | None of the time |
| q0058_0008 | 1.00 | All of the time |
|  | 2.00 | Most of the time |
|  | 3.00 | Some of the time |
|  | 4.00 | A little of the time |
|  | 5.00 | None of the time |
| q0058_0009 | 1.00 | All of the time |
|  | 2.00 | Most of the time |
|  | 3.00 | Some of the time |
|  | 4.00 | A little of the time |
|  | 5.00 | None of the time |
| q0059 | 1.00 | All of the time |
|  | 2.00 | Most of the time |
|  | 3.00 | Some of the time |
|  | 4.00 | A little of the time |
|  | 5.00 | None of the time |
| q0060_0001 | 1.00 | Definitely true |
|  | 2.00 | Mostly true |
|  | 3.00 | Not sure |
|  | 4.00 | Mostly false |
|  | 5.00 | Definitely false |
| q0060_0002 | 1.00 | Definitely true |
|  | 2.00 | Mostly true |
|  | 3.00 | Not sure |
|  | 4.00 | Mostly false |
|  | 5.00 | Definitely false |
| q0060_0003 | 1.00 | Definitely true |
|  | 2.00 | Mostly true |
|  | 3.00 | Not sure |
|  | 4.00 | Mostly false |
|  | 5.00 | Definitely false |
| q0060_0004 | 1.00 | Definitely true |
|  | 2.00 | Mostly true |
|  | 3.00 | Not sure |
|  | 4.00 | Mostly false |
|  | 5.00 | Definitely false |
| Q67 (all subsets) | 1.00 | GP |
|  | 2.00 | Gyanecologist/Gyanecological surgeon |
|  | 3.00 | Physiotherapist (pelvic) |
|  | 4.00 | Psychologist/Psychiatrist |
|  | 5.00 | ED/Emergency room visit |
|  | 6.00 | Fertility specialist |
|  | 7.00 | Other specialist (ie Gastroenterologist, Endocrinologist ,Urologist) |
| q0069_0001_0001 | 1.00 | 0 |
|  | 2.00 | 1 |
|  | 3.00 | 2 |
|  | 4.00 | 3 |
|  | 5.00 | 4 |
|  | 6.00 | 5+ |
| q0069_0002_0001 | 1.00 | 0 |
|  | 2.00 | 1 |
|  | 3.00 | 2 |
|  | 4.00 | 3 |
|  | 5.00 | 4 |
|  | 6.00 | 5+ |
| q0069_0003_0001 | 1.00 | 0 |
|  | 2.00 | 1 |
|  | 3.00 | 2 |
|  | 4.00 | 3 |
|  | 5.00 | 4 |
|  | 6.00 | 5+ |
| q0069_0004_0001 | 1.00 | 0 |
|  | 2.00 | 1 |
|  | 3.00 | 2 |
|  | 4.00 | 3 |
|  | 5.00 | 4 |
|  | 6.00 | 5+ |
| q0069_0005_0001 | 1.00 | 0 |
|  | 2.00 | 1 |
|  | 3.00 | 2 |
|  | 4.00 | 3 |
|  | 5.00 | 4 |
|  | 6.00 | 5+ |
| q0069_0006_0001 | 1.00 | 0 |
|  | 2.00 | 1 |
|  | 3.00 | 2 |
|  | 4.00 | 3 |
|  | 5.00 | 4 |
|  | 6.00 | 5+ |
| q0069_0007_0001 | 1.00 | 0 |
|  | 2.00 | 1 |
|  | 3.00 | 2 |
|  | 4.00 | 3 |
|  | 5.00 | 4 |
|  | 6.00 | 5+ |
| q0069_0008_0001 | 1.00 | 0 |
|  | 2.00 | 1 |
|  | 3.00 | 2 |
|  | 4.00 | 3 |
|  | 5.00 | 4 |
|  | 6.00 | 5+ |
| q0069_0009_0001 | 1.00 | 0 |
|  | 2.00 | 1 |
|  | 3.00 | 2 |
|  | 4.00 | 3 |
|  | 5.00 | 4 |
|  | 6.00 | 5+ |
| Q76-80 (all subcatagories) | 1.0 | Acupuncture/TCM |
|  | 2.0 | Naturopathy |
|  | 3.0 | Chiropractor |
|  | 4.0 | Dietician or nutritionist |
|  | 5.0 | Other CAM therapist (ie homeopath or herbalist) |
|  | 6.0 | Yoga or pilates |
|  | 7.0 | Physiotherapist |
|  | 8.0 | Osteopath |
|  | 9.0 | Massage |
|  | 10.0 | Psychology |
| Q81-84  (all subcatagories) | 1.0 | Cleaning/Gardening/ cooking/ Housework |
|  | 2.0 | Childcare |
|  | 3.0 | Meals/Food provided by others |
|  | 4.0 | Transportation |
| q0088 | 1.00 | Yes |
|  | 2.00 | No |
|  | 3.00 | N/A - I'm not/haven't been employed in the last 12 months |
| q0089_0001 | 1.00 | Lost job (resigned/fired) |
| q0089_0002 | 1.00 | Changed job |
| q0089_0003 | 1.00 | Reduced work hours |
| q0089_0004 | 1.00 | Other (please specify) |
| q0091 | 1.00 | Yes |
|  | 2.00 | No |
| q0094 | 1.00 | Yes |
|  | 2.00 | No |
