# Supplementary material for: The cost of illness and economic burden of endometriosis and chronic pelvic pain in Australia: A national online survey
Source: PLoS One. 2019 Oct 10;14(10):e0223316. doi: 10.1371/journal.pone.0223316 (PMC6786587; doi:10.1371/journal.pone.0223316)
Supplement: S1 Table — (PDF) [file pone.0223316.s005.pdf]

Endometriosis

	Age range		18-24		25-30		31-38		39+		All ages - total	
Health	Primary		930	(597,1200)	672	(483,1051)	1,055	(597,1410)	835	(503,1076)	883	(708,1102)
	Secondary		768	(535,1075)	908	(618,1102)	1,067	(729,1198)	953	(672,1126)	935	(774,1059)
	Out-of-Pocket		769	(509,1123)	794	(580,1273)	908	(499,1328)	778	(449,1208)	822	(675,1110)
	Total		2,467	(1642,3400)	2,374	(1682,3428)	3,030	(1826,3938)	2,566	(1625,3410)	2,640	(2158,3271)
Productivity	Abesteeism	Initial Impact	3,374	(3194,3549)	4,161	(3959,4315)	3,994	(3818,4496)	2,604	(2333,2766)	3,647	(3359,3944)
		Multiplier	3,273	(2975,3885)	4,036	(3678,4280)	3,874	(3570,4024)	2,526	(2245,2751)	3,538	(3273,3735)
	Presenteeism	Initial Impact	5,228	(4833,5875)	6,060	(5585,6553)	7,609	(7299,8255)	4,343	(3624,4686)	6,058	(5823,6472)
		Multiplier	3,660	(3358,4011)	4,242	(3885,4473)	5,326	(4834,5637)	3,040	(2752,3332)	4,240	(3950,4525)
	Total		15,534	(14361,17321)	18,500	(17108,19622)	20,803	(19522,22414)	12,514	(10955,13536)	17,484	(16407,18678)
Carers	Paid		-	-	84	(51,126)	174	(111,238)	103	(26,157)	97	(37,136)
	In-kind		395	(191,602)	501	(238,738)	795	(530,1200)	1,091	(744,1318)	677	(395,1125)
	Total		395	(191,602)	585	(289,865)	968	(642,1438)	1,193	(771,1475)	774	(432,1262)
	Grand Total		18,396	(16387,21926)	21,460	(19320,24655)	24,802	(22521,28992)	16,273	(14097,19742)	20,898	(19393,23212)

Chronic Pelvic Pain (CPP)

	Age range		18-24		25-30		31-38		39+		Total	
Health	Primary		1,290	(938,1648)	565	(340,931)	883	(520,1088)	212	(110,539)	819	(625,1038)
	Secondary		1,516	(1105,1855)	1,912	(1573,2393)	1,987	(1544,2408)	2,273	(1880,2577)	1,896	(1534,2455)
	Out-of-Pocket		698	(457,902)	315	(156,579)	569	(327,886)	253	(126,551)	500	(367,740)
	Total		3,503	(2501,4406)	2,791	(2070,3904)	3,439	(2392,4383)	2,738	(2117,3668)	3,215	(2528,4234)
Productivity	Abesteeism	Initial Impact	1,233	(806,1573)	1,826	(872,2418)	2,640	(2,193)	1,555	(1051,1975)	1,976	(1574,3208)
		Multiplier	1,196	(872,1429)	1,771	(845,2312)	2,561	(2028,2959)	1,508	(1075,2046)	1,917	(1502,2600)
	Presenteeism	Initial Impact	3,896	(3430,4832)	3,348	(2753,3932)	6,894	(5771,7979)	5,772	(4996,6744)	5,233	(4664,6120)
		Multiplier	2,727	(2419,3828)	2,343	(1847,2714)	4,826	(4214,8953)	4,040	(3409,5301)	3,663	(2878,4530)
	Total		9,053	(5108,7835)	9,287	(7528,11663)	16,920	(6318,11378)	12,875	(14222,20086)	12,789	(10533,16068)
Carers	Paid		247	(68,471)	73	(28,163)	81	(25,135)	-	#VALUE!	108	(36,187)
	In-kind		-	-	136	(65,254)	1,827	(940,2699)	681	(328,1319)	858	(356,1311)
	Total		247	(68,471)	209	(94,417)	1,908	(965,2834)	681	(328,1319)	966	(392,1499)
	Grand Total		12,803	(10097,16542)	12,288	(8483,15700)	22,267	(17580,27305)	16,294	(12979,21056)	16,970	(13540,22193)
