# Supplementary material for: The cost of illness and economic burden of endometriosis and chronic pelvic pain in Australia: A national online survey
Source: PLoS One. 2019 Oct 10;14(10):e0223316. doi: 10.1371/journal.pone.0223316 (PMC6786587; doi:10.1371/journal.pone.0223316)
Supplement: S2 Table — (PDF) [file pone.0223316.s006.pdf]

	MINIMUM		MILD		MODERATE		SEVERE	
	**Int $**	**95% CI**	**Int $**	**95% CI**	**Int $**	**95% CI**	**Int $**	**95% CI**
**HEALTH**	1,342	(341,1804)	1,588	(1133,1939)	2,301	(1914,2640)	2,399	(2004,2687)
**OUT-OF-POCKET**	472	(120,877)	533	(326,806)	946	(624,1326)	923	(583,1255)
**PRODUCTIVITY**	1,553	(793,1977)	10,554	(7833,12522)	13,792	(11613,15291)	19,378	(17897,21036)
**CARER**	437	(97,751)	386	(179,740)	652	(387,945)	1,286	(1006,2631)
**TOTAL**	3,805	(1617,5410)	13,063	(9472,16009)	17,692	(14539,20203)	23,987	(21492,26744)
